# Multi-Faceted Characterization of a Novel LuxR-Repressible Promoter Library for *Escherichia coli*

**DOI:** 10.1371/journal.pone.0126264

**Published:** 2015-05-26

**Authors:** Susanna Zucca, Lorenzo Pasotti, Nicolò Politi, Michela Casanova, Giuliano Mazzini, Maria Gabriella Cusella De Angelis, Paolo Magni

**Affiliations:** 1 Dipartimento di Ingegneria Industriale e dell’Informazione, Università degli Studi di Pavia, Pavia, Italy; 2 Centro di Ingegneria Tissutale, Università degli Studi di Pavia, Pavia, Italy; 3 IGM-CNR, Università degli Studi di Pavia, Pavia, Italy; Niels Bohr Institute, DENMARK

## Abstract

The genetic elements regulating the natural quorum sensing (QS) networks of several microorganisms are widely used in synthetic biology to control the behaviour of single cells and engineered bacterial populations via ad-hoc constructed synthetic circuits. A number of novel engineering-inspired biological functions have been implemented and model systems have also been constructed to improve the knowledge on natural QS systems. Synthetic QS-based parts, such as promoters, have been reported in literature, to provide biological components with functions that are not present in nature, like modified induction logic or activation/repression by additional molecules. In this work, a library of promoters that can be repressed by the LuxR protein in presence of the QS autoinducer N-3-oxohexanoyl-L-homoserine lactone (AHL) was reported for *Escherichia coli*, to expand the toolkit of genetic parts that can be used to engineer novel synthetic QS-based systems. The library was constructed via polymerase chain reaction with highly constrained degenerate oligonucleotides, designed according to the consensus -35 and -10 sequences of a previously reported constitutive promoter library of graded strength, to maximize the probability of obtaining functional clones. All the promoters have a *lux box* between the -35 and -10 regions, to implement a LuxR-repressible behaviour. Twelve unique library members of graded strength (about 100-fold activity range) were selected to form the final library and they were characterized in several genetic contexts, such as in different plasmids, via different reporter genes, in presence of a LuxR expression cassette in different positions and in response to different AHL concentrations. The new obtained regulatory parts and corresponding data can be exploited by synthetic biologists to implement an artificial AHL-dependent repression of transcription in genetic circuits. The target transcriptional activity can be selected among the available library members to meet the design specifications of the biological system.

## Background

Quorum sensing (QS) is a cell-to-cell communication mechanism exploited by Gram-negative and Gram-positive bacteria to implement a coordinated and density-dependent control of gene expression [1–4]. The expressed genes may have disparate functions in different bacteria, including virulence factors, biofilm formation and bioluminescence [1, 5]. Bacteria synthesize a signalling molecule, which diffuses across the cell membranes and, at sufficiently high bacterial densities, it activates a signalling cascade to induce coordinated gene expression in the population. One of the most studied QS systems is the LuxR/LuxI system, regulating bioluminescence in *Vibrio fischeri* [6]. Here, the density-dependent behaviour is regulated by two genes: *luxI*, encoding for an autoinducer synthase, responsible for the production of the small signalling molecule N-3-oxohexanoyl-L-homoserine lactone (AHL), and *luxR*, encoding for the LuxR transcription factor that is activated by AHL. The activated complex LuxR-AHL binds a DNA domain called *lux box*, located upstream of the P_lux_ promoter, thus activating the transcription of downstream genes, involved in bioluminescence, in a concentration-dependent fashion [6].

QS elements have been widely used in synthetic biology to control the behaviour of single cells and bacterial populations via ad-hoc constructed gene networks. Such elements have been exploited to construct exogenously inducible input devices to regulate gene expression in several synthetic devices, such as a lysis device [7], logic gates and functions [8, 9] and different model systems used to study the predictability of biological systems design [10, 11]. QS elements have been included in circuits implementing complex engineering-inspired functions, such as pattern formation [12], pulse generation [13], population density control [14], density-dependent gene expression devices [15, 16], logic functions, including XOR, NOR, EQUAL gates, multiplexer and demultiplexer functions and layered logic circuits [9, 17–19], synchronized oscillators [20, 21], as well as the creation of a synthetic predator-prey ecosystem of two bacterial populations [22]. These studies aimed to test the feasibility of a bottom-up design approach or to investigate the complexity that can be reached in the realization of customized systems with predictable behaviour.

Synthetic circuits have also been constructed with the aim of improving the knowledge on the behaviour of natural QS systems [23, 24]. Natural QS elements have been rearranged to study density-dependent quantitative gene expression among different network topologies [25]. Copy number-related phenomena on the steady-state input-output characteristic of QS circuits have been investigated [26]. A generic metric has been defined to quantify the ‘sensing potential’ and the system dynamics in comparable units among different QS systems [27]; the same research group also quantitatively studied the dynamics of bacterial populations secreting a costly but beneficial enzyme to characterize the robustness of such circuit architecture [28]. The specificity of different QS systems from different organisms has been studied to quantify the cross-talk effects in the *lux* and *las* systems [29].

In order to improve the knowledge on the quantitative behaviour of QS elements and to expand the list of available parts, novel synthetic components were designed and built up starting from known regulatory elements. Promoters represent key regulatory elements in gene network design. The design of their response logic and the tuning of their activity in a rigorous fashion is necessary in order to meet the specifications derived from circuit design.

Constitutive active variants of the LuxR transcription factor were constructed by mutagenesis or truncation to create an AHL-independent LuxR-inducible promoter, functioning as an amplifier that can be re-used in different synthetic networks [30].

QS-based synthetic promoters have been designed to be responsive either to multiple autoinducer molecules [17] or to additional exogenous or endogenous stimuli [13, 31, 32]. Promoters have also been designed to show inverted or modified activation logic [31–34].

Many efforts have been carried out to create promoters able to sense two or more inputs. Tamsir et al. constructed tandem promoters composed by the sequence of two promoters with the same orientation, behaving as an OR gate with two QS signalling molecules (or other molecules) as inputs [17], while Basu et al. constructed a hybrid promoter inducible by AHL and repressible by the cI protein, obtaining a novel regulatory element that was used to implement a transient activation of gene expression, mimicking a ‘pulse’ generation [13]. Shong and Collins engineered the natural P_esaR_ promoter, repressible by the EsaR-AHL complex, to obtain a set of QS-inducible systems with disparate gene expression levels, regulatory range, AHL sensitivity and timing [32]. They also designed two synthetic promoters that behave as AND gates, requiring both AHL and another molecule (IPTG or ATc) as inputs [33]. The two works of Shong and Collins relied on the change of position of the binding sites for transcriptional regulators. The set of available QS elements has been further expanded by Adams et al., who used directed evolution to isolate *lsrR* genes (starting from the *E*. *coli* master QS regulator) able to show novel activation and repression logic [35].

Cox et al. randomly combined different transcription factor binding sites (e.g., *lux box*, AraC, LacI and TetR operator sites) in the same promoter, in the distal, core or proximal position, obtaining a library of mixed-regulated promoters, responding to one or more stimuli and exhibiting different activation logics [31].

Egland and Greenberg [34] reported the design of a LuxR-AHL repressible promoter, by inserting a *lux box* between (and partially overlapping) the *E*. *coli* consensus -35 (TTGACA) and -10 (TATAAT) promoter regions. This promoter exhibited an inverted logic, compared to the wild type P_lux_ promoter, being repressed by the LuxR-AHL complex.

There is no accordance between the findings of Cox et al. [31] and Egland and Greenberg [34], since the same LuxR-AHL repressible promoter has been studied and different logic responses were observed. While Egland and Greenberg measured a significant decrease of the activity for increasing AHL concentrations, Cox et al. did not observe a meaningful variation in these contexts. However, a different plasmid measurement system and a different experimental setup have been used in the two studies.

Serious efforts have been performed in the development of predictive tools to relate promoter strength to the DNA sequence, but no strategy has shown to fully capture this cryptic relation [36]. In fact, many factors have been identified as crucial features in the determination of promoter strength. Besides the -35 and -10 regions, other elements have shown to significantly affect promoter strength, such as spacers and sequences flanking the -35 and -10 regions [37, 38]. For this reason, libraries of synthetic promoters have been constructed, so that synthetic biologists can individuate the part with the most promising features and employ it in the network to obtain the desired behaviour.

Several techniques for libraries generation have been exploited, such as random mutagenesis of existing promoters via error-prone PCR [39], PCR with degenerate primers performed via Taq polymerase [40], degenerate oligonucleotide annealing and fill-in via the Klenow polymerase [41] or shuffling of existing parts, representing the distal, core or proximal promoter regions, de-novo synthesized with compatible overhangs [31].

**Fig 1:**
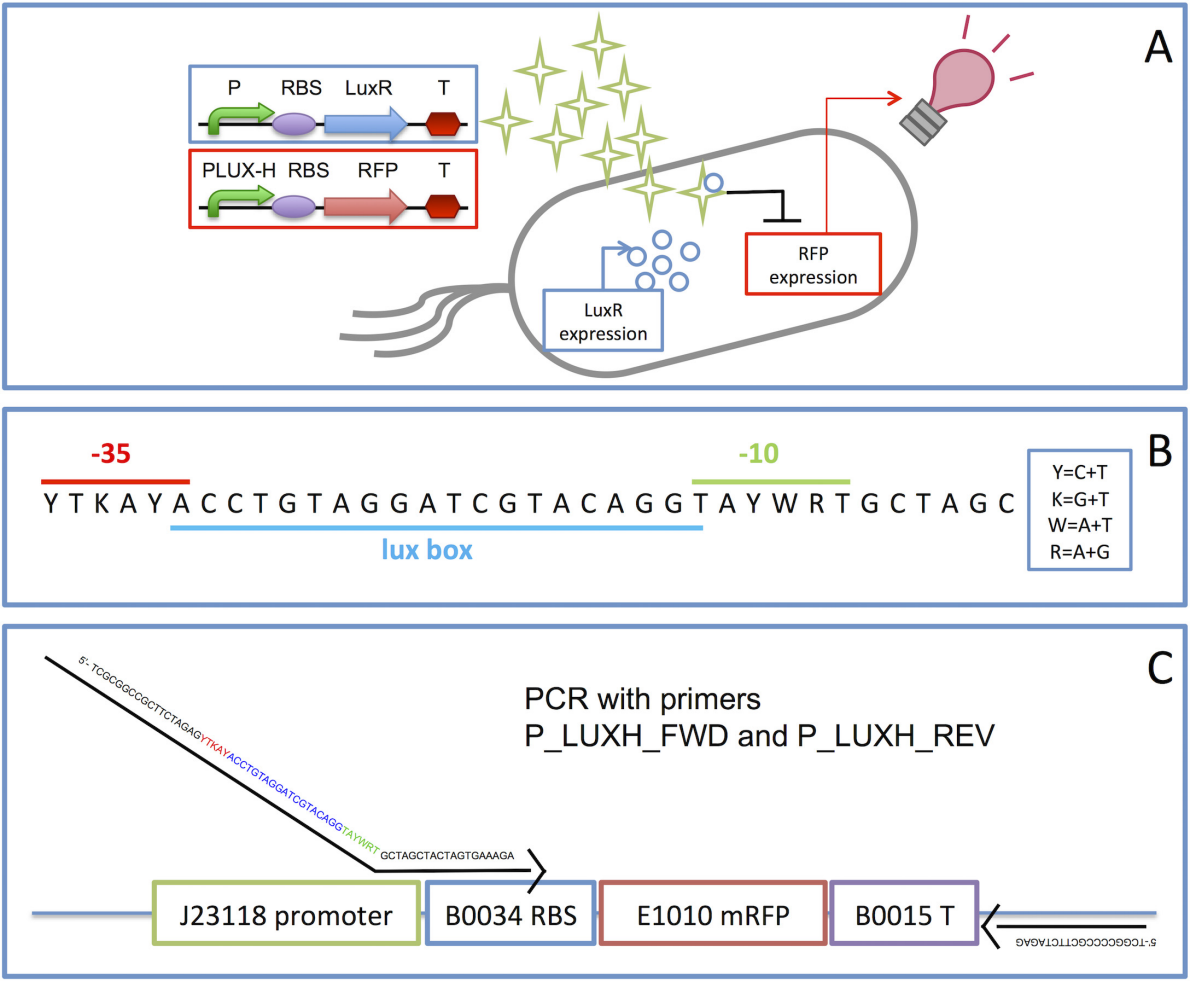
Structure and schematic behaviour of the LuxR-repressible promoters. A) Schematic representation of the working principle of the repressible promoters. In the blue and red boxes the two synthetic networks representing the constitutive LuxR expression cassette and the LuxR-regulated RFP expression cassette are shown, respectively (curved green arrows represent promoters, purple ovals represent RBSs, coloured arrows represent genes and red hexagons represent transcriptional terminators). Green stars represent the AHL inducer molecules, diffusing through cell membrane and binding the LuxR transcription factor (blue circles). This complex represses the expression of RFP. B) The consensus sequence of the library of LuxR-repressible promoters is reported, according to the IUPAC nucleotide code (reported in the box on the right). -35 (red line), -10 (green line) and lux box (blue line) regions are highlighted. C) Generation method of the library via PCR with degenerate primers. The template is BBa_J23118 in the BBa_J61002 plasmid. In every box, the BioBrick code of the corresponding part is reported. The forward primer P_LUXH_FWD anneals with the template in a region falling between promoter and RBS and has a floating tail containing the degenerate sequence (the -35, lux box and -10 regions are highlighted in red, blue and green, respectively), while the reverse primer P_LUXH_REV anneals with the sequence downstream of the transcriptional terminator.

In this work, we report the generation of a library of LuxR-repressible promoters (see Fig 1A), inspired by the promoter designed by Egland and Greenberg [34], via PCR with degenerate primers. The -35 and -10 regions have been varied to obtain a library of members with graded strength. With the intent to increase the probability of finding promoters with non-null and diverse strength, the degenerate nucleotides of the -35 and -10 regions were chosen according to an already existing promoter collection of working elements (the Anderson constitutive promoter collection [42]). A multi-faceted characterization has been carried out via two different reporter genes (encoding the Red Fluorescent Protein—RFP—and the Green Fluorescent Protein—GFP) in two different copy number contexts, in presence or absence of the LuxR transcription factor and via population or single-cell measurements.

The obtained synthetic promoters, constituting the library, are available in the Registry of Standard Biological Parts (Registry) with BioBrick codes from BBa_J107100 to BBa_J107111 and all the documentation and experience has been shared in this repository [43].

## Materials and Methods

### Strains, plasmids and media

TOP10 *E*. *coli* (Invitrogen) were used for parts cloning and tests. Plasmids were propagated in recombinant strains grown in selective LB medium [44]. TOP10 bacteria were heat-shock transformed according to the manufacturer’s protocol. Ampicillin (100 mg/l), chloramphenicol (12.5 mg/l) and kanamycin (25 mg/l) were added as required. Long-term bacterial stocks were prepared for all the recombinant cultures by mixing 750 μl of a saturated culture with 250 μl of glycerol 80% and stored at -80°C.

Apart from the newly synthesized promoters, whose construction is described below, all the parts and plasmids used in this study were assembled from basic or composite parts from the Registry 2009 or 2010 DNA Distribution [43], according to the BioBrick Standard Assembly procedure [43, 45, 46].

DNA-modifying enzymes were purchased from Roche. DNA purification kits were purchased from Macherey-Nagel. All parts were verified through sequencing, performed via the BMR Genomics DNA analysis service (Padova, Italy). AHL (#K3007, Sigma Aldrich) was dissolved in deionized water to prepare a 2 mM stock, routinely stored at -20°C.

Table 1 lists the basic and composite parts used in this study. Among all the selected random promoters (parts 1–90), the ones included in the final library have been submitted to the Registry with the codes BBa_J107101-BBa_J107110, as well as the BBa_J107100 promoter which has been obtained via a non-random procedure (see below). A second PCR round was carried out and parts 91–135 were generated and screened to find repressible promoters with activity falling in a range not covered by the other previously identified members: one promoter was selected and submitted to the Registry with the code BBa_J107111.

**Table 1:** Parts and plasmid vectors used in this study.

Name	Description
**PARTS**	
BBa_S03119	LuxR expression cassette driven by the strong promoter PL_tetO1_
BBa_I13507	Promoter-less RFP expression cassette including RBS BBa_B0034, BBa_E1010 RFP reporter gene and BBa_B0015 terminator
BBa_E0240	Promoter-less GFP expression cassette including RBS BBa_B0032, BBa_E0040 RFP reporter gene and BBa_B0015 terminator
BBa_J107100*	LuxR-repressible promoter library member (similar to BBa_R0061) constructed via oligonucleotide annealing
BBa_J107101-BBa_J107110*	LuxR-repressible promoter library members, obtained via PCR with degenerate primers
BBa_J107111*	LuxR-repressible promoter, obtained via the second round of PCR with degenerate primers
BBa_J23100	Promoter from the Anderson library
BBa_J23101	Promoter from the Anderson library
BBa_J23102	Promoter from the Anderson library
BBa_J23104	Promoter from the Anderson library
BBa_J23105	Promoter from the Anderson library
BBa_J23106	Promoter from the Anderson library
BBa_J23110	Promoter from the Anderson library
BBa_J23114	Promoter from the Anderson library
BBa_J23116	Promoter from the Anderson library
BBa_J23117	Promoter from the Anderson library
BBa_J23118	Promoter from the Anderson library
BBa_J23119	Promoter from the Anderson library
BBa_R0011	P_LlacO1_ promoter
BBa_I14032	P_lacIQ_ promoter
BBa_R0051	P_R_ promoter
1–90*	Random promoters obtained by PCR, selected in the low-copy (1–44) and high-copy (45–90) contexts
91–135*	Random promoters obtained via the second round of PCR, selected in the low-copy context
**VECTORS**	
BBa_J61002	High copy number ampicillin-resistant vector, identical to pSB1A2, but with an mRFP1 expression system (BBa_I13507) between SpeI and PstI
pSB4C5	Low copy number, chloramphenicol-resistant vector
pSB3K3	Medium copy number, kanamycin-resistant vector
pSB1A2	High copy number, ampicillin-resistant vector

All the parts are from Registry of Standard Biological Parts, with the exception of the asterisk-marked parts, which have been constructed in this study.

The resulting library includes 12 members. Table 1 does not report all the promoter measurement constructs, whose description is provided below.

### Library construction

#### Consensus sequence determination

The LuxR-repressible promoters are characterized by the presence of a 20-bp DNA domain (*lux box*) for the binding of the LuxR-AHL activated complex in the core region, between the -35 and -10 sequences [31, 34]. In order to obtain a graded-strength library, the -35 and -10 sequences were varied, according to a consensus sequence, while the *lux box* was maintained unaltered.

The Anderson constitutive promoter collection [42] includes elements that are widely used for the construction of synthetic circuits. Among its members, BBa_J23119 is the ‘consensus promoter’ that has the -35 (TTGACA) and -10 (TATAAT) sequences corresponding to the *E*. *coli* promoter consensus sequences, like the LuxR-repressible promoter designed by Egland and Greenberg [34]. The Anderson collection was considered as the starting point to define the consensus sequence of the members of the library. The -35 and -10 regions were analyzed and the consensus was determined according to the following criteria: only the promoters where the last nucleotide of the -35 is A and the first nucleotide of the -10 is T (corresponding to the first and last base of the *lux box*, respectively) were included in the consensus determination. The resulting consensus sequence is reported in Fig 1B and potentially enables the generation of 64 different promoters.

#### Construction of the BBa_J107100 LuxR-repressible promoter

The BBa_R0061 part from the Registry was initially considered to be included as a member of the library. It is composed by the same -35 (TTGACA) and -10 (TATAAT) regions as the BBa_J23119 promoter of the Anderson collection and the *lux box* sequence is present in the ‘core’ position, to provide the AHL-repressible behaviour. However, this part cannot be readily assembled upstream of a gene expression system composed by an RBS-gene-Terminator sequence because the promoter ends with the -10 region. For this reason, the transcription start site (+1) would lie after the beginning of the RBS sequence and the assembly of a spacer between promoter and RBS would be required to properly express the downstream gene. To overcome this problem, a new LuxR-repressible promoter was constructed via oligonucleotide annealing, according to the Anderson library sequence and with the -35 and -10 sequences of BBa_R0061 and BBa_J23119. Two oligonucleotides (sense—J107100_FWD and anti-sense—J107100_REV) were designed according to the structure of the BBa_J23119 promoter (see the sequences in Table 2). The oligonucleotides were synthesized (Sigma Aldrich) with phosphorylated 5’-ends resembling an EcoRI (forward oligonucleotide) and a SpeI (anti-sense oligonucleotide) digestion of a BioBrick part. The annealing reaction was performed in the annealing buffer (100 mM NaCl, 10 mM Tris-HCl, pH 7.4) with the sense and the anti-sense oligonucleotides at the final concentration of 100 μM. The annealing reaction mix was heated at 100°C for 5 minutes, and then allowed to cool slowly at room temperature. The resulting part, named BBa_J107100, was then ligated in the EcoRI/SpeI-digested BBa_J61002 high-copy plasmid, which has the RBS-RFP-Terminator construct (identical to BBa_I13507) between the SpeI and PstI sites. The sequence between BBa_J107100 and the RBS is not the BioBrick scar ‘TACTAGAG’, but ‘TACTAGTG’. For the quantitative characterization experiments, this part was then EcoRI/PstI-digested and assembled in an EcoRI/PstI-digested pSB4C5 low-copy plasmid (chloramphenicol-resistant, with a pSC101 replication origin).

**Table 2:** List of oligonucleotides used to generate the library members and to sequence-verify all the plasmids.

Primer	Sequence
J107100_FWD	5′*phos AATTCGCGGCCGCTTCTAGAGTTGACACCTGTAGGATCGTACAGGTATAATGCTAGCTA 3′
J107100_REV	5′*phos ACTAGTAGCTAGCATTATACCTGTACGATCCTACAGGTGTCAACTCTAGAAGCGGCCGCG 3′
P_LUXH_FWD	5′ TCGCGGCCGCTTCTAGAGYTKAYACCTGTAGGATCGTACAGGTAYWRTGCTAGCTACTAGTGAAAGA 3′
P_LUXH_REV	5′ AGCGAGGAAGCCTGCAGC 3′
P_LIB_FWD	5′ TCGCGGCCGCTTCTAGAG 3′
VF2	5′ TGCCACCTGACGTCTAAGAA 3′
VR	5′ ATTACCGCCTTTGAGTGAGC 3′

5′*phos indicates that the 5'-end is phosphorylated. The sequence is indicated according to the IUPAC nucleotide code.

#### Construction of the library of LuxR-repressible promoters via PCR with degenerate primers

The BBa_J23118 promoter from the Anderson promoter collection [42] in the BBa_J61002 plasmid was used as a template for library generation via PCR. The forward and reverse primers (P_LUXH_FWD and P_LUXH_REV, respectively) were designed according to the following criteria: i) the last portion of the forward primer anneals with exactly 20 nucleotides of the template (in the BBa_J23118 expression system, see Fig 1C) after the last degenerate nucleotide of the primer; ii) the degenerate floating tail of the forward primer matches the library consensus sequence, composed by the -35 and -10 regions consensus (determined by the sequences of the members of the Anderson promoter collection) and the *lux box* in the core region, overlapping for one nucleotide with both the -35 and -10 sequences (see consensus sequence in Fig 1B); iii) the forward primer contains part of the BioBrick prefix, including the XbaI site, to allow part cloning according to the BioBrick Standard Assembly [46]; iv) the reverse primer anneals with the PstI-containing region of BBa_J61002, so that the RFP coding sequence is included in the PCR amplicon (Fig 1C). The primer sequences are reported in Table 2. A first PCR cycle was performed using the Phusion Hot Start Flex DNA Polymerase (New England Biolabs), according to the manufacturer’s protocol, on BBa_J23118 template with P_LUXH_FWD and P_LUXH_REV primers. The PCR cycles were programmed as follows: i) 30 s at 98°C; ii) a temperature cycle sequence of 10 s at 98°C—30 s at 52°C—30 s at 72°C, repeated 5 times; iii) a temperature cycle sequence of 10 s at 98°C—45 s at 72°C, repeated 30 times; iv) 10 min at 72°C. The PCR product was run on a 1% agarose gel and the correct band (908 bp) was extracted and purified (Extract II kit, Macherey-Nagel).

This product was then used as a template for a second PCR reaction (3 min at 98°C; 10 s at 98°C—30 s at 63°C—30 s at 72°C, repeated 5 times; 10 min at 72°C) with P_LIB_FWD and P_LUXH_REV primers (see Table 2), to remove the annealing between heterologous strands of promoter sequences [40]. The resulting fragment was purified as before, XbaI/PstI-digested and ligated in the XbaI/PstI-digested pSB1A2 high-copy plasmid (ampicillin-resistant, with mutated pMB1 replication origin) and in the XbaI/PstI-digested pSB4C5 plasmid. The resulting plasmids have an RFP expression device (identical to BBa_I13507) downstream of the promoter, with the non-BioBrick 'TACTAGTG' DNA junction between them.

The obtained parts in both the low- and high-copy plasmids were transformed in *E*. *coli* TOP10 and the grown colonies were selected according to a visual inspection of RFP expression: transformation plates with colonies bearing low-copy plasmids were used to try to select phenotypes only for very strong promoters and not to give a metabolic burden impairing cell growth, while plates with colonies bearing high-copy plasmids should allow the selection of medium to weak promoters. The selected clones were assayed as described in the next section both in the constitutive and regulated conditions. Then, only the clones with detectable fluorescence and which showed repression upon AHL induction were considered. All the promising candidates in the high-copy condition were then transferred in the low-copy plasmid pSB4C5 and all the resulting promoters in the low-copy condition were sequence-verified. Promoters with unique sequences were selected as members of the library. The members of the LuxR-repressible promoter family obtained via the first PCR round with degenerate primers are named BBa_J1071xx, with xx = 01,02,…,10.

### Library characterization

#### Construction of the measurement systems

**Fig 2:**
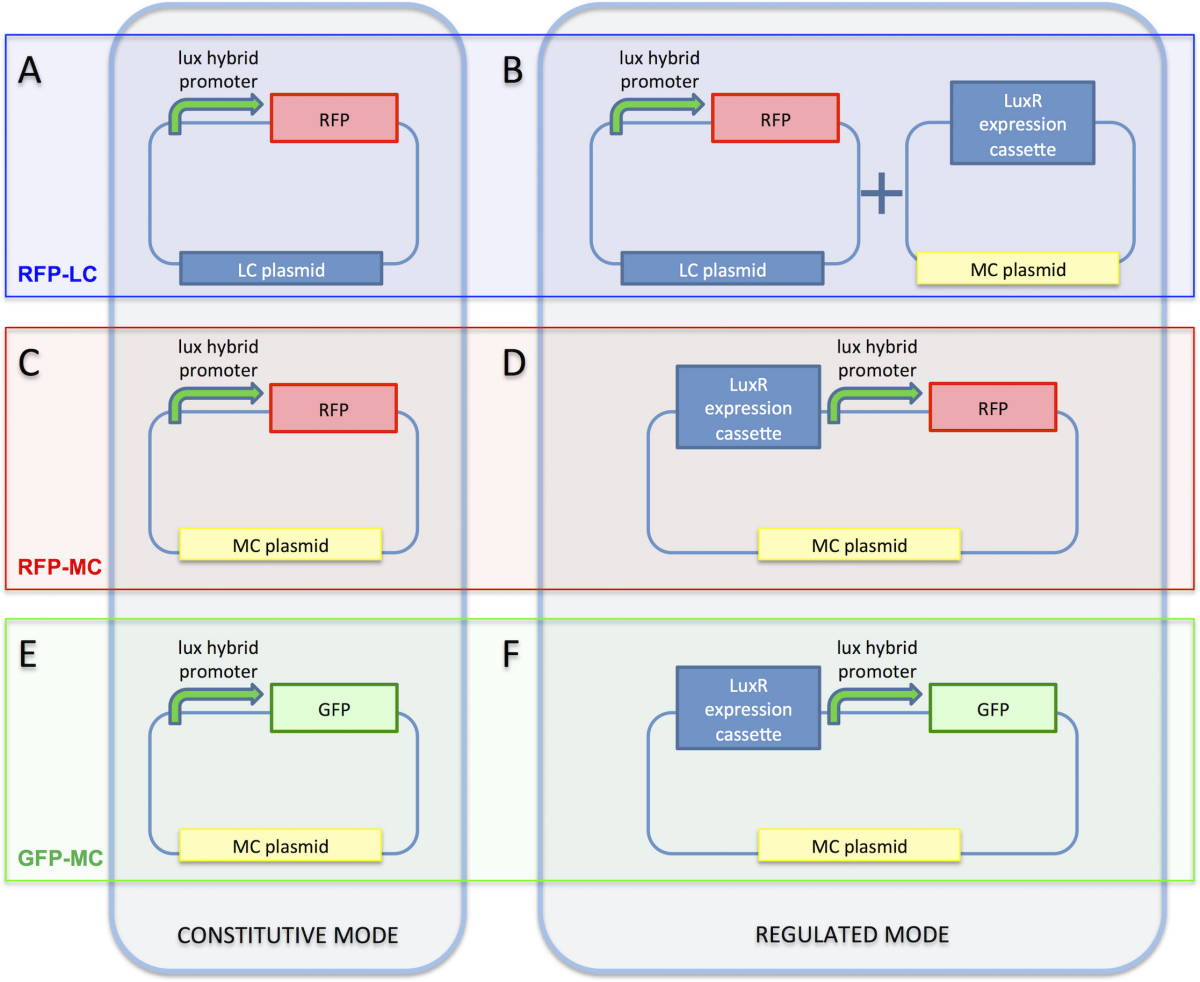
Characterization contexts for the library of LuxR-repressible promoters. The 'constitutive mode' indicates the configuration in which the LuxR expression cassette is absent, while the 'regulated mode' indicates the context in which the LuxR expression cassette is present. A-B panels show the RFP-LC context, in constitutive and regulated mode, respectively; C-D represent the RFP-MC context, in constitutive and regulated mode, respectively; E-F represent the GFP-MC context, in constitutive and regulated mode, respectively. LC and MC plasmids are the pSB4C5 low-copy plasmid and pSB3K3 medium-copy plasmid, respectively. The '+' symbol in panel B indicates that both plasmids are present in the cell.

The library members were characterized in different contexts. The ‘constitutive’ mode indicates the design that does not include a LuxR expression cassette (Fig 2), while the ‘regulated’ mode indicates the design in which a LuxR expression cassette is present in the circuit.

The constitutive version of the library, obtained as described above, is shown in Fig 2A. The library is hosted by the pSB4C5 low-copy plasmid and drives the RFP expression system (BBa_I13507) composed by the RBS BBa_B0034, the RFP coding sequence BBa_E1010 and the transcriptional terminator BBa_B0015. This configuration is named ‘RFP-LC’.

The LuxR-regulated behaviour of promoters was studied by co-transforming *E*. *coli* TOP10 cells bearing the RFP-LC library members with the pLuxR-MC plasmid, which is the pSB3K3 plasmid containing the LuxR expression cassette BBa_S03119 as insert. This configuration is named ‘RFP-LC-LUXR’ (Fig 2B).

The RFP-LC promoters with RFP expression cassette were EcoRI/PstI-digested and assembled in the EcoRI/PstI-digested pSB3K3, thus obtaining the constitutive medium-copy context (RFP-MC) (Fig 2C). Like in RFP-LC, the DNA junction between promoter and RFP expression system is non-standard ('TACTAGTG'). To create the regulated version of this context, the LuxR expression cassette was assembled upstream of the promoters by EcoRI/XbaI-digesting the RFP-MC plasmids and inserting the EcoRI/SpeI-digested insert of pLuxR-MC. This context is named ‘RFP-MC-LUXR’ (Fig 2D).

GFP was assayed as a different reporter. The BBa_E0240 GFP generator (composed by RBS BBa_B0032, GFP BBa_E0040 and transcriptional terminator BBa_B0015) was XbaI/PstI-digested and inserted in both the SpeI/PstI-digested RFP-MC and RFP-MC-LUXR constructs (thus replacing the RFP expression system with a GFP expression system). These contexts are named GFP-MC and GFP-MC-LUXR, respectively (Fig 2E and 2F). Here, the scar between the promoter and the RBS is the standard BioBrick scar ‘TACTAGAG’.

The Anderson promoter library members were used to investigate an experimentally observed and previously uncharacterized repressible behaviour of BBa_J23101 by the LuxR-AHL complex. All the sequence-verified Anderson promoters available in the Registry (listed in Table 1, indicated by the BioBrick code BBa_J231xx) in the BBa_J61002 plasmid were transferred in the low copy plasmid pSB4C5 by EcoRI/PstI digestion (thus transferring the promoter and its downstream RFP expression cassette, with the non-standard DNA junction 'TACTAGTG' between them) and assayed in presence of pLuxR-MC. The same tests were also performed with the P_LlacO1_, P_lacIQ_ and P_R_ promoters, instead of the members of the Anderson library (in this case with the standard DNA scar 'TACTAGAG').

**Fig 3:**
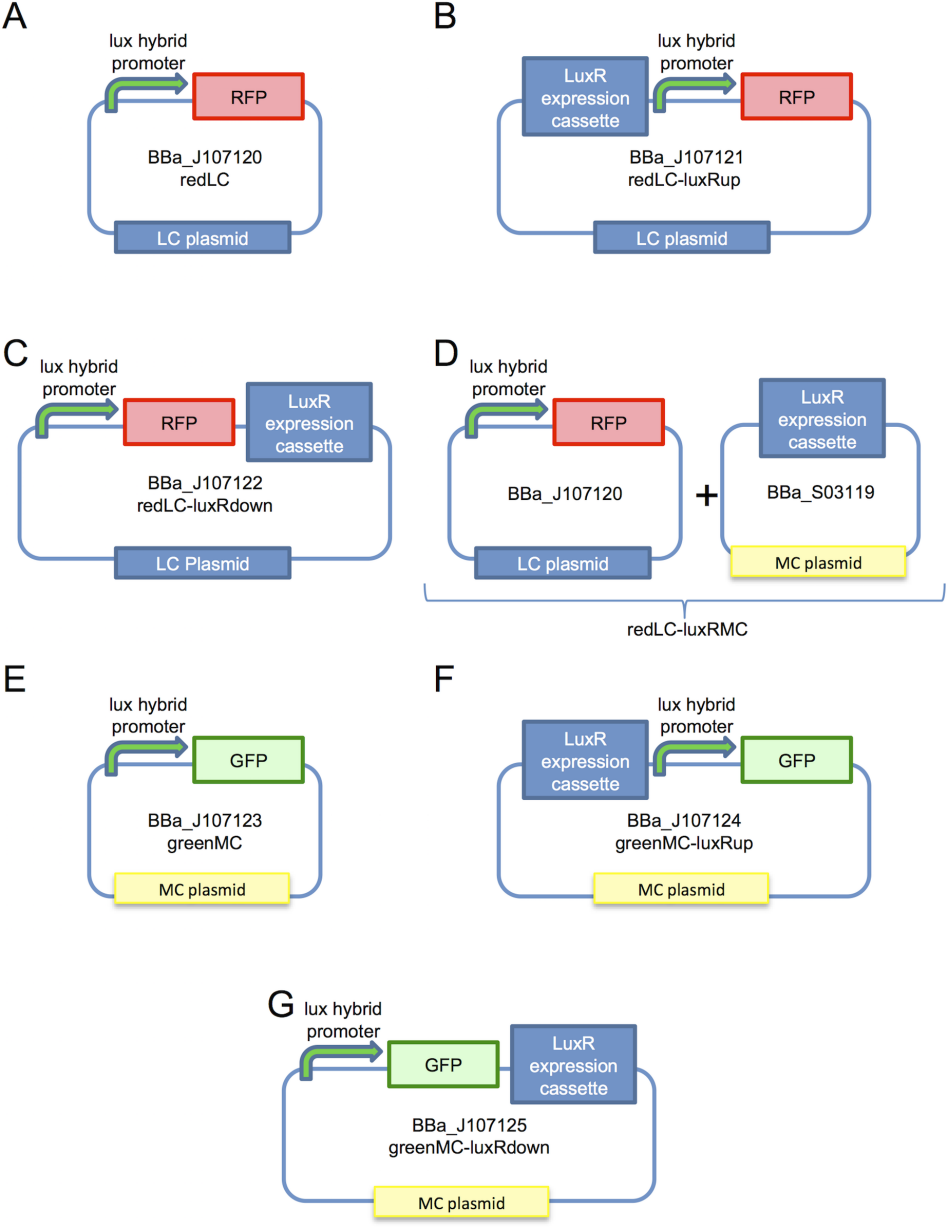
Configurations studied to test the 'position effect' of the LuxR-expression cassette. Here, the LuxR-repressible promoter BBa_J107100 (green curved arrow), driving the expression of RFP (red box) or GFP (green box), has been assembled in different configurations: in low-copy pSB4C5 (LC) plasmid with the RFP reporter gene A) without the LuxR-expression cassette, B) assembled downstream or C) upstream of the LuxR-expression cassette, and D) co-transformed with the LuxR-expression medium-copy plasmid pLuxR-MC; in medium copy pSB3K3 (MC) plasmid with the GFP reporter gene E) without the LuxR-expression cassette, F) assembled downstream or G) upstream of the LuxR-expression cassette. The BioBrick codes are indicated for each part and a name is provided for each configuration. The '+' symbol in panel D indicates that both plasmids are co-transformed in the cell.

The effect of physical position of BBa_S03119 (i.e., upstream or downstream of the promoter-driven reporter expression cassette, or borne by a different plasmid) on the activity of the BBa_J107100 promoter was assayed by constructing redLC (BBa_J107120), redLC-luxRup (BBa_J107121), redLC-luxRdown (BBa_J107122), redLC-luxRMC (BBa_J107120 co-transformed with BBa_S03119), greenMC (BBa_J107123), greenMC-luxRup (BBa_J107124) and greenMC-luxRdown (BBa_J107125) parts. These constructs were submitted to the Registry and are described in Fig 3.

#### Library characterization via population-based measurements

Glycerol stocks were streaked on LB agar plates supplemented with the proper antibiotics. Plates were incubated at 37°C for 16 hours. Single colonies were picked and used to inoculate 1 ml of selective M9 supplemented medium (11.28 g/l M9 salts, 1 mM thiamine hydrochloride, 2 mM MgSO_4_, 0.1 mM CaCl_2_, 0.2% casamino acids and 0.4% glycerol), incubated at 37°C, 220 rpm for 16 hours. Cultures were 100-fold diluted in 200 μl of selective M9 in a 96-well microplate. When required, 2 μl of properly diluted AHL were added into the plate wells to reach the desired final inducer concentration. Deionized water was used to prepare the AHL dilutions. No water was added to the wells where AHL-inducible cultures were assayed without inducer. The plate was then incubated in the Infinite F200 microplate reader (Tecan) and the following kinetic cycle, programmed with the i-control software (Tecan), was used to assay the cultures: every 5 min, 15 s linear shaking (3 mm amplitude), 5 s wait, optical density at 600 nm (OD) measurement, fluorescence measurement. The total duration of the kinetic cycle was about 18 hours. RFP detection was carried out with the 535/620 nm filters for excitation/emission and acquisition gain as appropriate, ranging from 50 to 100. GFP-expressing cells were assayed through the same procedure, but using the 485/540 nm filters for fluorescence acquisition. In every experiment, 200 μl of M9 supplemented medium and a non-fluorescent TOP10 culture were also included to measure the background of OD and fluorescence, respectively [47].

#### Library characterization via single-cell measurements

Recombinant strains expressing GFP were inoculated and grown as described in the previous section. Grown cultures were 100-fold diluted in 2 ml of selective M9 supplemented medium. AHL was added, when required, at the final concentration of 0 M, 5 nM and 100 nM (fully activated, half-repressed and totally repressed configurations, respectively, see Results section). After 2 to 3 hours from the induction, cells were properly diluted in sterile PBS (supplemented with 1 mg/l of gentamicin to stop bacterial protein production [48]) and used for flow cytometric analysis.

Cells were analyzed through a Partec PAS II flow cytometer equipped with an argon ion laser using the 488 nm blue line for excitation. Fluorescence emission was collected in FL1 by means of a 515–545 nm band pass filter. At least 100,000 events were collected and stored for each sample. A non-fluorescent TOP10 culture was always included to measure the background fluorescence.

#### Library expansion

After the characterization step for all the 11 library members obtained so far, a new PCR round was performed with degenerate primers to search for promoters with constitutive activity between 0.7 and 2 RPUs, since this range was not covered by the generated members. The same procedure described above was carried out, except that the template promoter was BBa_J23116 instead of BBa_J23118, and the resulting PCR amplicons were ligated only in the low-copy vector pSB4C5.

### Data analysis

#### Repression curves computation

Raw absorbance and fluorescence time series were processed to obtain doubling time, average RFP and GFP synthesis rate per cell (*S*_*cell*_) and Relative Promoter Units (RPUs) during the exponential growth phase for each culture. OD, RFP and GFP were background-subtracted as previously described [10, 11]. Exponential growth phase limits were set to OD values of 0.05 and 0.2, respectively, identified by visual inspection of the linear region of the *ln*(*OD*) time series in our experiments. The slope *m* of this line was computed as the average of the numeric time derivative of *ln*(OD) in the exponential phase to yield the cell growth rate. Doubling time was computed as *ln*(2)/*m*.

A signal proportional to the RFP or GFP synthesis rate per cell was computed as the numeric time derivative of fluorescence, divided by OD over time. This signal was averaged over the exponential growth phase to obtain the mean *S*_*cell*_ value, expressed in Arbitrary Units of RFP or GFP per minute per cell (AU min^-1^ cell^-1^). Relative units, to express the activity of promoters in standardized measurement units, were computed as *RPU*_*x*_ = *S*_*cell*,*x*_/*S*_*cell*,*ref*_, where *x* is the culture of interest and *ref* is the culture bearing the standard reference promoter (BBa_J23101) with RFP or GFP, in the same copy number conditions and with identical measurement system (and DNA junction between promoter and downstream reporter expression system) as the culture of interest, and acquired with the same gain factor.

Repression curves were fitted with the Hill equation $Y=\delta +\frac{V_{\text{MAX}}}{1+{\left(\frac{A}{K_{M}}\right)}^{\eta }}$, where δ is the basic activity in the fully repressed state, K_M_ is the switch point of the Hill function, δ+V_MAX_ is the maximum activity in absence of AHL and η is the Hill coefficient. Y is the RPU of the system and 'A' is the AHL concentration in the culture medium.

The coefficient of variation (CV) of the estimated parameters was computed via a Monte Carlo simulation method. In particular, 10,000 synthetic datasets were generated by adding Gaussian noise (with zero mean and variance = ${\hat{\sigma }}^{2}$, defined below) to the identified Hill function model. Noise variance was computed as ${\hat{\sigma }}^{2}=\frac{RSS}{N-k}$, where RSS is the sum of squared residuals, N is the number of original experimental data and k is the number of parameters (k = 4). Each dataset was fitted with the same model and initial parameters as the original data. The CV was calculated from the obtained parameter distributions.

All data analyses were performed via Matlab R2010a software (Mathworks, Natick, MA, USA). Least square optimization was performed using the Matlab *lsqnonlin* routine.

#### Flow cytometric data processing

Flow cytometric data from single-cell experiments, in FCS format, were exported and processed using an FCS data reader package (fca_readfcs script, Laszlo Balkay [48]) via Matlab. The log-binned values of forward scatter (FSC), side scatter (SSC) and fluorescence (FL1) are integer values in the range 0–4095. Events with FL1 = 0 were discarded [48]. Log-binned FSC, SSC and FL1 values were converted into linear-scale values and a proper FSC/SSC-gating was performed to remove undesired events. In all the experiments, more than 20,000 events survived after the described processing. Finally, the FL1 arithmetic mean of the non-fluorescent culture was subtracted from the FL1 values of fluorescent cultures in order to remove background.

RPUs were calculated as described above, by using the background-subtracted fluorescence values instead of *S*_*cell*_. Cell-to-cell variability (in terms of CV) was also calculated for every experiment.

Although the originally proposed formulas for RPU computation include the growth rate [47], here it was not considered (in both population and single-cell measurements), since no reasonable difference could be detected in the measured growth rates (see S1 Fig) and the conclusions of the paper are not affected by the inclusion of these values (data not shown).

#### Statistical analysis

Statistical analysis was performed as reported in [11]: the ANOVA test was performed to evaluate the statistical difference among the mean RPU values in a group of conditions. Individual homoscedastic t-tests were carried out for the groups that showed a statistically significant difference, to compare the mean values of the group members and identify statistically different sub-groups. The CV was computed among the mean values of these sub-groups. Individual t-tests were corrected with the Bonferroni correction for multiple comparisons. The final CV within a group was computed on the mean values of the statistically different sub-groups, which were averaged. If the ANOVA test found a significant difference, but multiple comparisons showed no evident sub-groups, the CV was computed on all the mean activities among the group. All the coefficients of variation were corrected for small samples, according to the formula $CV_{corrected}=CV\cdot \left(1+\frac{1}{4N}\right)$, where N is the number of samples. The statistical analysis was performed via Matlab.

## Results and Discussion

### Library construction

All the promoters were initially present in low-copy (LC) or high-copy (HC) vector with an RFP expression cassette downstream, thus enabling quantitative measurements via reporter protein. A total of 90 clones were selected and screened by measuring constitutive RFP expression and repression capability in the regulated mode (promoters 1–90). At the end of this screening, the functional promoters in high-copy were transferred into the pSB4C5 low-copy vector.

A total of 28 candidates, all in low-copy plasmid, were obtained and sequenced via the VF2 and VR primers (Table 2). 10 unique promoter sequences, different from BBa_J107100, were identified and selected as library members. For this reason, considering BBa_J107100, the resulting library contains 11 different promoters with non-null activity in the constitutive mode and a repressible behaviour in presence of LuxR-AHL. Their sequence is reported in Table 3.

**Table 3:** Sequences of the promoters belonging to the LuxR-repressible promoter library.

Name in this study	BioBrick code	-35	Core sequence	-10	Proximal sequence
2	BBa_J107101	TTGAC	ACCTGTAGGATCGTACAGGT	A**C**A**G**T	GCTAGC
20	BBa_J107102	TTGA**T**	ACCTGTAGGATCGTACAG**G**T	ATAGT	GCTAGC
35	BBa_J107103	TTGAC	ACCTGTAGGATCGTACAGGT	AT**T**AT	GCTAGC
39	BBa_J107104	TTGA**T**	ACCTGTAGGATCGTACAGGT	A**C**AAT	GCTAGC
44	BBa_J107105	TTGAC	ACCTGTAGGATCGTACAGGT	A**CTG**T	GCTAGC
47	BBa_J107106	TTGAC	ACCTGTAGGATCGTACAGGT	A**C**AAT	GCTAGC
51	BBa_J107107	**C**T**T**AC	ACCTGTAGGATCGTACAGGT	A**C**AAT	GCTAGC
55	BBa_J107108	**C**TGAC	ACCTGTAGGATCGTACAGGT	A**C**AAT	GCTAGC
62	BBa_J107109	TTGAC	ACCTGTAGGATCGTACAGGT	ATA**G**T	GCTAGC
79	BBa_J107110	TTGAC	ACCTGTAGGATCGTACAGGT	AT**TG**T	GCTAGC
122	BBa_J107111	TTGAC	ACCTGTAGGATCGTACAGGT	A**CT**AT	GCTAGC
J107100	BBa_J107100	TTGAC	ACCTGTAGGATCGTACAGGT	ATAAT	GCTAGC

The sequence of BBa_J107100 is reported in the last row and the mutated bases are in bold type and underlined.

The screening procedure for library generation was based on fluorescence measurements in single clones without replicates, in heterogeneous plasmid measurement systems (LC and HC). In order to fully characterize the library performance, the promoters were then tested in different contexts and with a suitable number of biological replicates.

### Library characterization

All the 11 members of the library of repressible promoters have been assembled in the six contexts represented in Fig 2.

**Fig 4:**
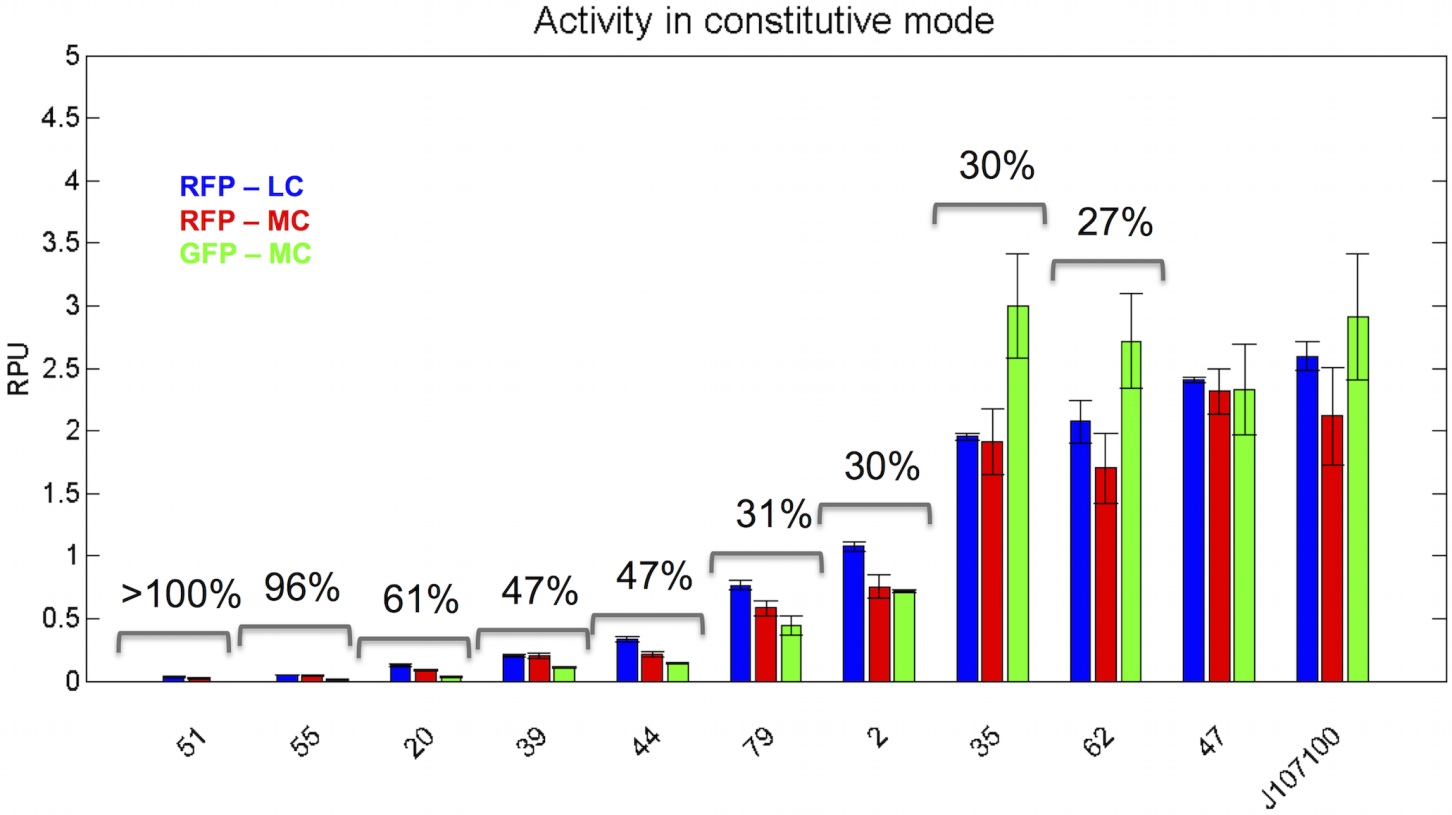
Constitutive activity of the LuxR-repressible promoters with three different measurement systems. Bars represent the average of at least three experimental replicates and error bars represent the 95% confidence intervals. CV is reported when a statistically significant difference was measured among the three experimental conditions (p-value<0.05, ANOVA).

The constitutive activity is shown in Fig 4, using the 3 different measurements systems illustrated in Fig 2A, 2C and 2E. Considering RFP activity in the context of Fig 2A (RFP-LC), constitutive promoters show a graded activity and span 92-fold activity range, with a mean spacing of about 44% [39]. A similar activity range and mean spacing is obtained for the RFP-MC context (117-fold and 45%, respectively), while such range could not be computed for the GFP-MC context, since there are promoters with activity under detection limits, which result in null activities. This problem is due to the higher fluorescence background in GFP acquisitions.

The strongest promoters have RPU activities comparable to the widely used P_LtetO1_ promoter (according to unpublished experiments of our group). For the promoters with activities that are significantly different among the three measurement systems (p-value<0.05, ANOVA), CV was computed. Promoters with RPU values greater than 0.1 always have a CV lower than 61%, while CV is much higher for the two weakest promoters, probably because of the high noise levels affecting measurements. Considering library members with RPU>0.1, 7 out of 9 promoters show a statistical difference among the tested conditions. CV entities are consistent with previous studies of our group on context-dependent variability of individual promoters [11, 49], where the maximum CV was about 30% for promoters with RPU>0.4, while they are reasonably contained when compared with other studies [38, 50, 51].

**Fig 5:**
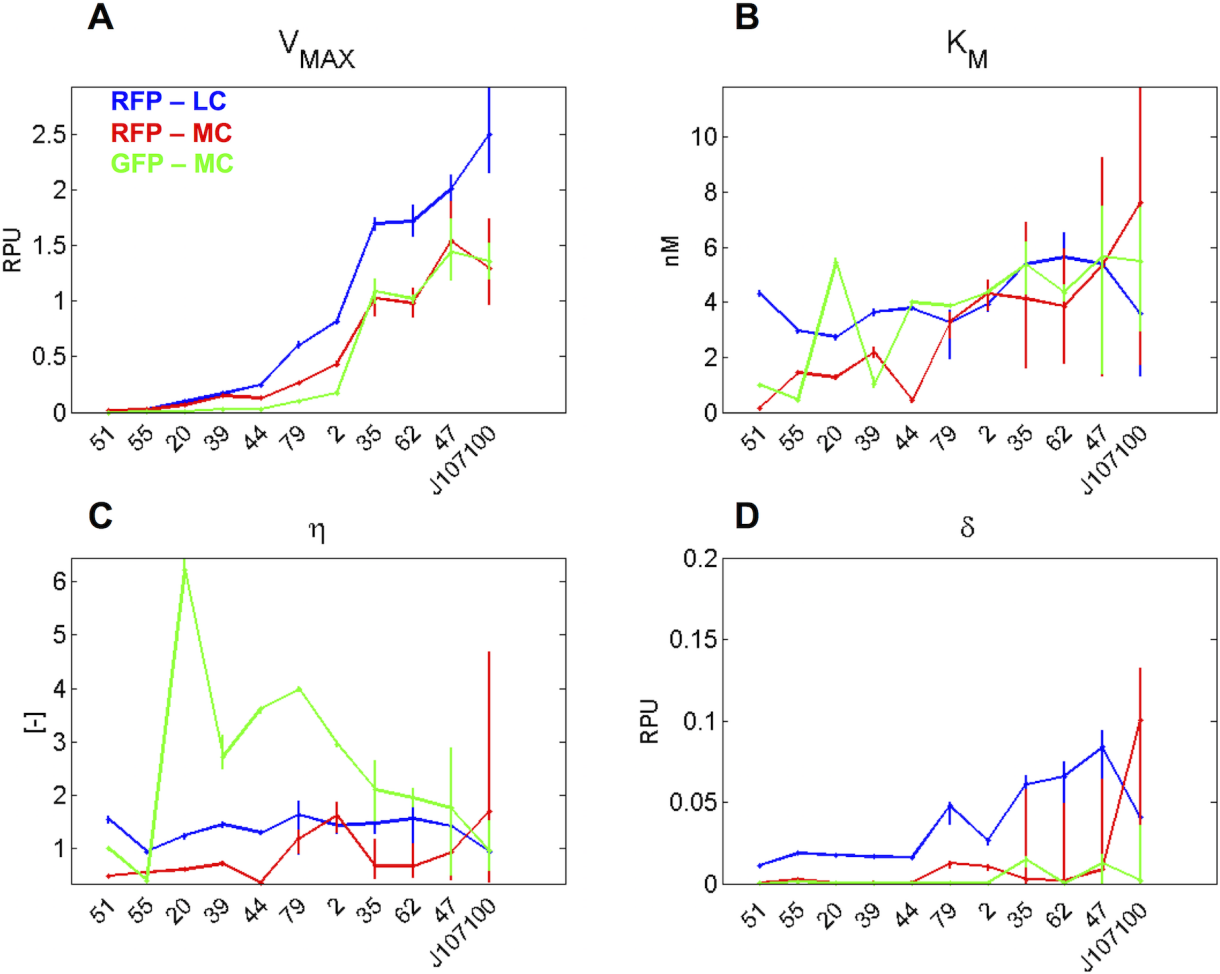
Hill function parameter values obtained by fitting the repression curves of the promoters in the regulated mode. Three different measurement systems are reported, as indicated on the figure.

The regulated mode was then tested and repression curves were fitted with a Hill function, as described in the Methods section. The individual fitted curves, reported in S2 Fig, indicate that all the promoters show an AHL-dependent repression, which can be fitted with a Hill curve. A summary of the estimated parameter values is shown in Fig 5, for the three different measurement configurations. As expected, V_MAX_ is highly correlated with the constitutive strength of promoters (compare Fig 5A with Fig 4), while all the other parameters do not show significant trends as a function of maximum strength or context. The switch point of the Hill curves is between 0.15 and 7.6 nM (see Fig 5B), although the majority of promoters have a K_M_ value between 1 and 6 nM, with the values outside this range probably deriving from fitting problems in clones with low activity (see S2 Fig). The obtained K_M_ values are highly consistent with the half-induction AHL concentrations obtained in previous studies (1–3 nM) involving devices with the wild type P_lux_ inducible promoter, where the *luxR* gene was expressed at high levels by the P_LtetO1_ promoter (like in this study) in the MG1655 strain [10, 26] or by the IPTG-induced P_LlacO1_ promoter in the MG1655-Z1 strain [52]. The Hill coefficient is below 2 in all the conditions except in some clones in the GFP-MC context (Fig 5C). As before, these high Hill coefficient values are reasonably due to fitting problems, which result in very steep curves (probably caused by measurement noise in the GFP signal, which is characterized by a higher background than the RFP signal), as shown in S2 Fig for clones with very low activity. Finally, the activity of all the promoters in the off-state is much lower than the respective V_MAX_ values (Fig 5D).

Importantly, as shown in Fig 5A, the V_MAX_ value has a different trend between low copy and medium copy contexts: in fact, promoters characterized in low-copy plasmid have a V_MAX_ which is on average 2-fold higher than in the medium-copy biological measurement systems (considering conditions with RPU>0.1). This might be due to cell overburdening or nonlinear saturation phenomena occurring in the cell, related to copy number increase [26]. However, it is worth noting that promoters characterized in the constitutive condition did not show such difference and they have an activity consistent with the V_MAX_ values in the regulated low-copy context (compare Fig 5A, blue data points, with Fig 4). This effect may also be caused by the different sequences upstream of promoters (see Fig 2B, 2D and 2F), which can affect their transcriptional activity. This phenomenon will be investigated below.

In summary, in the studied contexts, copy number showed a major impact on library strength in presence of LuxR, causing on average 2-fold variations in maximum strength, while the Hill-related parameters (switch point, Hill coefficient and leakage activity) did not show a significant context-dependent variation. On the other hand, the use of GFP or RFP as reporter system did not result in important differences in promoter activity.

**Fig 6:**
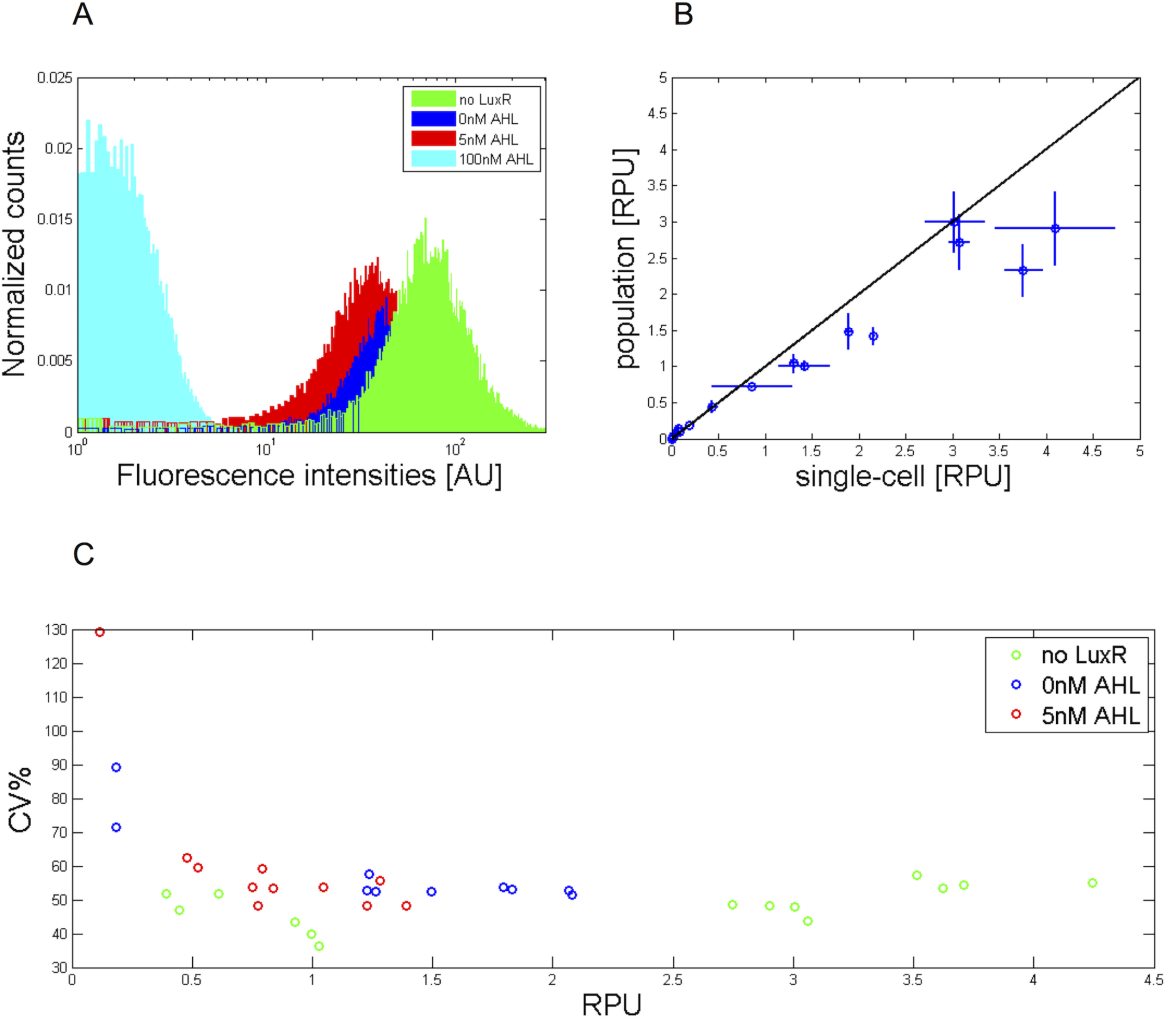
Single-cell measurements of the activity of the LuxR-repressible promoters. A) Representative fluorescence distributions for BBa_J107100 promoter assayed in constitutive mode (green) and regulated mode with no (blue), intermediate (red) and full (cyan) induction. B) Promoter activity obtained via population-based measurements versus single-cell measurements. Blue circles represent average RPU values, horizontal and vertical error bars represent the 95% confidence intervals. The solid black line is the y = x line. C) Cell-to-cell variability, in terms of CV, is reported as a function of RPU.

Single-cell measurements have been performed in order to evaluate the library activity with a different measurement system, and to evaluate if fluorescence distributions in cell populations have a unimodal behaviour. Library members have been assayed in constitutive and regulated modes, in the GFP-MC context. Regulated modes were assayed in the full (100 nM), intermediate (5 nM) and no induction conditions. Fluorescence distributions are shown in Fig 6A for a representative culture. No clones showed bimodal distributions, confirming that population behaviour is homogeneous and that population measurements are suitable to characterize such devices. Population-based and single-cell measurements also provided highly comparable values (see Fig 6B). For cultures with average RPU>0.1, cell-to-cell variability, in terms of CV of the fluorescence distribution, was computed and CV values as a function of RPUs are reported in Fig 6C. It is worth noting that CV values do not depend on promoter strength and their average value is about 55%. This observation is important for all the applications where noise contribution needs to be taken into account, such as the study of the behaviour of interconnected module and noise propagation in a genetic network.

### Library expansion

The 11-member library obtained so far shows an uncovered constitutive activity range between about 0.7 and 2 RPUs (see Fig 4). Promoters with such activities are commonly used in synthetic circuits. For this reason, a second PCR round was performed to search for LuxR-repressible promoters that could fill this gap. Forty-five random promoters were screened (parts 91–135) and one new promoter with the desired features was selected (part 122, see Table 1) and submitted to the Registry as BBa_J107111. This promoter was characterized in the RFP-LC context via population-based measurements and results in the constitutive mode are shown in S3A Fig. The AHL-dependent repression curve of this promoter in the regulated mode is shown in S3B Fig, together with its estimated Hill parameter values. Analogously to the other promoters, the activity in the constitutive and regulated mode without AHL is similar. The Hill parameter values are highly consistent with the ones of the other library members. The activity mean spacing of the library including this new member decreases from 44% to 39%. A summary of the sequence of all the final 12 library members is reported in S4 Fig.

### Effects of LuxR expression cassette on promoter activity

In order to investigate the effects of the presence of flanking sequences on promoter activity and to evaluate their impact in different configurations, seven recombinant strains have been constructed (see Fig 3), by varying the relative positions between the LuxR expression cassette and a representative LuxR-AHL repressible RFP expression cassette driven by the BBa_J107100 promoter. The redLC, redLC-luxRup and redLC-luxRdown devices have the RFP expression cassette in LC plasmid and the LuxR expression cassette is absent (Fig 3A), upstream (Fig 3B) or downstream (Fig 3C) of the LuxR-repressible promoter, respectively. The redLC-luxRMC condition, with BBa_J107120 co-transformed with pLuxR-MC, is also studied to consider a source of LuxR on a different plasmid (Fig 3D). The greenMC, greenMC-luxRup and greenMC-luxRdown devices have the GFP expression cassette in MC plasmid and the LuxR expression cassette is absent (Fig 3E), upstream (Fig 3F) or downstream (Fig 3G) of the promoter, respectively. The described devices can elucidate if the decreased promoter activity observed in the RFP-MC and GFP-MC regulated contexts is due to sequence-dependent effects or cell overburdening.

**Fig 7:**
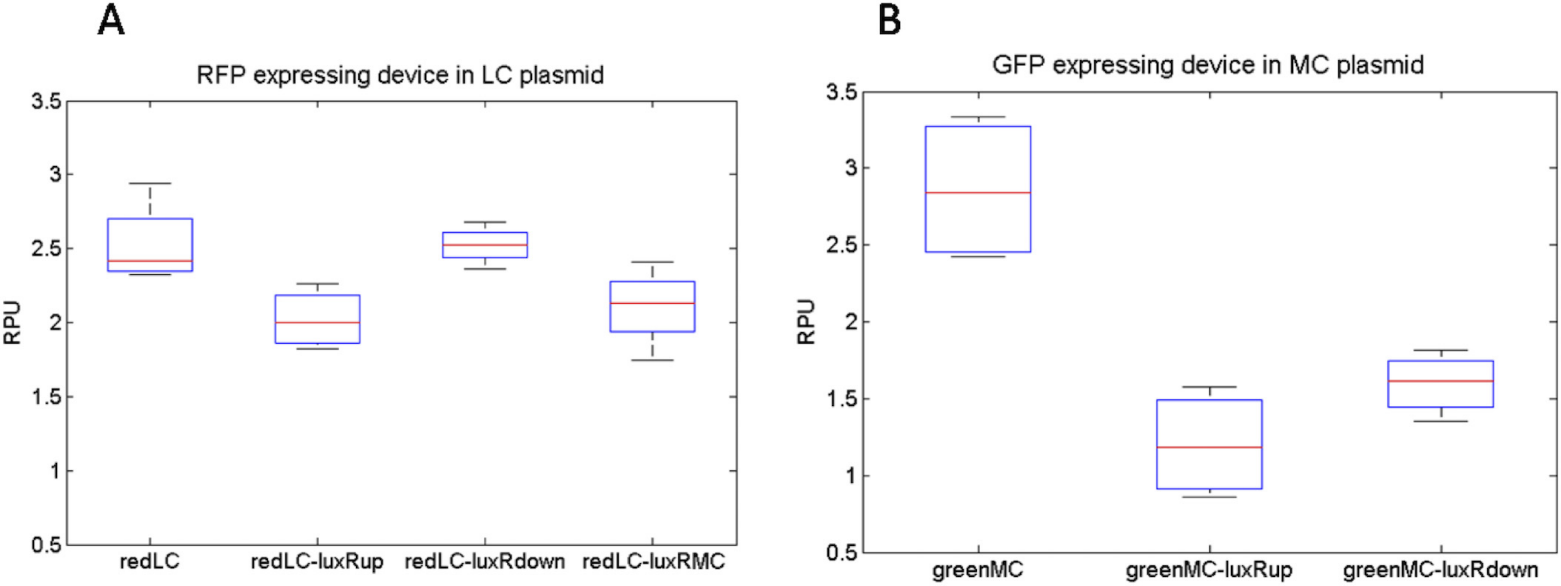
Boxplots showing the LuxR-repressible J107100 promoter activity in presence of a LuxR expression cassette in different positions. The description of the tested contexts is provided in the text.

The activity of the LuxR-repressible promoter was measured in absence of AHL (maximum activity) as described in the Methods section and the results were used to perform ANOVA tests. Significant differences in promoter activities have been observed (p-value = 0.0141) between the four strains expressing RFP in LC plasmid (see Fig 7A), however post-hoc comparisons did not show a clear separation. The CV was computed between all the data available in the three experimental conditions, resulting to be only 12%. This CV value is low and consistent with previous results obtained in our lab while studying context-dependent activity of two promoters placed in the same cell in two independent expression cassettes [11]. This result demonstrates that the presence of the LuxR expression cassette upstream of the promoter is not sufficient to explain the activity decrease in MC context compared to LC context.

In the GFP expressing strains, ANOVA test showed that a significant difference is present between promoter activities (p-value = 0.0003) and post-hoc comparisons showed a clear separation between the device without the LuxR expression cassette (greenMC) and the ones with the LuxR expression cassette, upstream or downstream of the promoter (greenMC-luxRup and greenMC-luxRdown). In the latter case, promoter activity is reduced by about 2-fold. This result suggests that the presence of an additional expression cassette in MC plasmid can be responsible of the overload of cell machinery, thus causing a diminished promoter activity, even though no effect on the doubling times was observed (see S1 Fig, compare the bars of constitutive and regulated mode for both the RFP-MC and GFP-MC contexts).

### Characterization of the AHL-repressible behaviour of BBa_J231xx promoters

While performing preliminary control experiments, we found that the BBa_J23101 promoter unexpectedly showed repression in presence of LuxR and AHL.

This phenomenon has been further investigated by characterizing all the available promoters from the Anderson library, in LC plasmids in presence of LuxR from the pLuxR-MC plasmid. Other promoters were also included in this analysis: P_LlacO1_ (BBa_R0011 promoter from the Registry in the BBa_J107010 measurement system, expressing RFP), P_lacIQ_ (BBa_I14032 promoter from the Registry, assayed in the BBa_J107012 measurement system, expressing RFP) and P_R_ (BBa_R0051 promoter assayed in the BBa_I763007 measurement system, expressing RFP) in order to validate their behaviour in presence of the LuxR-AHL complex.

The results of a preliminary test are reported in S5A Fig. Here, the activity in presence (green bars) or absence (blue bars) of 20 μM AHL, when LuxR expression cassette was present, was measured for all the tested promoters. Only BBa_J23101, BBa_J23102 and BBa_J23104 showed a significant repression, while in absence of a LuxR source (pLuxR-MC) repression did not occur (data not shown). For the promoters showing repressible behaviour, dose-response curves were studied. Results are reported in S5B Fig and show a decreasing activity for increasing AHL values. The maximum observed repression in presence of a very high AHL concentration (20 μM) was about 78%, 28% and 49% for BBa_J23101, BBa_J23102 and BBa_J23104, respectively.

To our knowledge, such phenomena were not reported in literature, and additional efforts should be required to elucidate the underlying mechanism, especially when such promoters are used in complex gene networks where their specific constitutive activity is crucial to enable the correct functioning. This effect has been documented in the Experience section of the BBa_J23101, BBa_J23102 and BBa_J23104 Registry pages.

## Conclusions

Naturally-occurring cell-to-cell communication mechanisms are widely used as a source of genetic elements to engineer biological systems that show disparate complex functions [24]. In order to meet design specifications for the system of interest, synthetic biologists have either used the natural members of the available toolkit of components or used newly constructed elements. In the latter case, the collection of parts involved in QS has been expanded to engineer a range of unnatural behaviours for promoters and transcriptional regulators.

At the state of the art of synthetic biology, the cryptic relation occurring between promoter nucleotide sequence and strength has not been fully captured, thereby limiting the availability of predictive tools to drive the de-novo design process. The creation of libraries of graded-strength promoters can face such limitations by providing synthetic biologists with a catalogue of parts to choose the one with the desired behaviour.

In this work, we report the design, realization and characterization of a novel library of LuxR-repressible promoters for *E*. *coli*. The LuxR-repressible behaviour depends on the presence of a *lux box* (naturally occurring in the P_lux_ promoter of *Vibrio fischeri*) in the core sequence of all the promoters, while the graded strength is obtained by varying the nucleotide sequences of the -35 and -10 regions, according to the consensus determined from an existing constitutive promoter library (the Anderson promoter collection [42]).

The construction of promoters with the *lux box* in the core sequence was inspired by the work of Egland and Greenberg [34], who validated the repressible behaviour of a promoter with this design for the first time, although in literature there is also a report where such design did not result in a functional promoter [31]. Consistently with the work of Egland and Greenberg, in our study almost all of the screened candidates resulted in a LuxR-repressible behaviour in presence of AHL for different -35 and -10 regions.

The availability of catalogues of quantitatively characterized parts can support the rational design of biological systems by enabling the choice of suitable components for the correct functioning of the target system, which can be studied, for instance, via mathematical modelling tools. A central problem in synthetic biology is the context-dependent variability of parts performance when they are used in different circuits or conditions. For this reason, the characterization of parts activity in different contexts and measured via different instruments can strongly support the re-use of genetic parts and could increase the probability of obtaining the expected quantitative behaviour in the designed system. To further support this process, standardized units are also crucial for the re-use of components in different laboratories where different measurement equipment is available. In light of the mentioned requirements, we carried out a multi-faceted characterization of our new library of components by reporting promoter activities in RPU. Two reporter measurement systems (RFP and GFP) were tested downstream of the promoters, in plasmids that are maintained at two different per-cell copy number, and measurements were carried out *in vivo* via two different instruments: a microplate reader to perform population-based acquisitions and a flow cytometer to provide single-cell data. Since all the obtained promoters can be used as constitutive or repressible elements, their activity in absence or presence of LuxR was measured, respectively, to provide data on the use of the promoters in both contexts.

The final LuxR-repressible promoter library described in this work is composed by 12 members spanning an activity range from about 0.03 to about 3 RPUs and with about 42% mean spacing between the activities of adjacent members. All of them can be tightly repressed by the LuxR-AHL complex, as demonstrated by dose-response experiments.

One of the 12 final members (BBa_J107100) did not result from the random screening process, but it was independently designed according to the -35 and -10 sequences of BBa_R0061, a LuxR-repressible promoter present in the Registry, that was designed according to the promoter constructed by Egland and Greenberg [34]. Because BBa_R0061 is not readily usable in the assembly of synthetic constructs due to a too short nucleotide sequence, BBa_J107100 was constructed and included in the library. Another promoter of the 12-member library (BBa_J107111) was obtained via a second PCR round aimed at searching for promoters with activity falling in the range 0.7–2 RPUs, which is not covered by the other library members but is commonly found in promoters used for synthetic circuit design.

Promoter activity in the constitutive mode, i.e., without a source of LuxR, showed a reasonably contained context-dependent variability among the three biological measurement systems used, with a CV not greater than 31% for promoter members with activity higher than 0.4 RPU. This variability value indicates the expected activity deviation that can be observed when re-using the promoter library members in a new context, such as a gene regulatory network or an expression cassette for a different gene. Although we studied different parts with different measurement systems, the CV among the tested contexts was consistent with our previous works, where promoters with a similar RPU range was investigated [11, 49]. In this study we tested the promoters upstream of RFP (BBa_I13507, in low- and medium-copy plasmid) and GFP (BBa_E0240, in medium-copy plasmid) reporter systems in TOP10; in [11] we obtained a CV of 22% when testing a set of promoters upstream of RFP (BBa_I13507 and BBa_K516032, which have different RBSs upstream of the RFP coding sequence) and GFP (BBa_E0240) reporter systems, all in low-copy plasmid in TOP10; finally, in [49] we obtained a CV of 31% when testing a set of promoters upstream of RFP (BBa_I13507) in low-copy plasmid or in two different genomic positions (Φ80 phage integration locus and aspA locus) in MG1655. The context-dependent maximum variability of the individual promoter activity obtained in this study is lower than the one observed in other reports [38, 50, 51].

For promoters in the regulated mode, i.e., in presence of LuxR, the AHL-RPU data were fitted with a Hill function and the resulting parameters can be used to summarize the quantitative behaviour of the repressible devices. Although some of the promoters showed fitting problems due to their low activity, the overall distribution of the estimated parameters did not have a clear strength- and context-dependent behaviour, except the maximum activity of promoters (V_MAX_ parameter) which was consistent with their constitutive activity, as expected, but was also dependent on the copy number context. In fact, V_MAX_ in medium-copy (with both RFP and GFP reporter systems) was 2-fold lower than in the low-copy context, measured via RFP. The K_M_ parameter was highly consistent with the one of the wild type P_lux_ promoter when characterized in presence of the same LuxR generator device [10, 26] or a similar one [52].

To investigate this activity difference, additional genetic contexts were tested, by evaluating the activity of one representative library member (BBa_J107100) in low- (with RFP) and medium- (with GFP) copy contexts with the LuxR expression cassette in different positions: upstream or downstream in the same plasmid, in a different plasmid (only for the low-copy with RFP context), or absent. The results clearly demonstrated that the above observed activity difference was due to the presence of two expression cassettes in a medium-copy plasmid, probably causing a metabolic overload in the host cell, while no significant upstream sequence-dependent effect could be detected when the LuxR expression cassette was present upstream of the reporter expression cassette driven by BBa_J107100.

Flow cytometry was used to measure the activity of promoters in one of the above tested contexts (GFP, medium-copy) via different measurement equipment. All the tested constructs showed a unimodal fluorescence distribution, whose arithmetic mean was highly consistent with the one obtained via population-based measurements. Single-cell data also gave the occasion to estimate cell-to-cell variability of clones. Excluding promoters with RPU<0.1, an average 55% CV was observed, independently from promoter activity, presence of LuxR expression cassette, and AHL addition. The quantification of cell-to-cell variability has an important impact on the bottom-up design of synthetic networks from basic modules, since it can be used to model and predict interconnection effects that cannot be trivially obtained from population-based measurements [53]. However, it is still unclear which are the features affecting cell-to-cell variability and the use of these constructs in different genetic, strain, environmental contexts could lead to variability values that deviate from the ones obtained in this study.

In addition, an undocumented LuxR-dependent repression of three members of the widely used Anderson promoter collection (BBa_J23101, BBa_J23102 and BBa_J23104) in presence of AHL was reported in this study. This repression entity was 78%, 28% and 49%, respectively, at 20 μM AHL and cannot be explained by cell growth variation, which is almost unaffected (data not shown). Other 9 tested members of the Anderson collection were unaffected, as well as the three popular P_lacIQ_, P_LlacO1_ and P_R_ promoters. This phenomenon should be taken into account when these parts are used as constitutive promoters in circuits containing the *luxR* gene and AHL is present, since unpredictable effects may occur, due to a non correct evaluation of their transcriptional strength. Although the maximum repression entity was obtained at 20 μM AHL, which is a very high concentration, it is worth noting that commonly used AHL levels (10 nM to 1 μM) also resulted in a non-null repression.

Considering the initial 11-member library construction process, the number of obtained members (10 unique clones of graded strength) is consistent with other works in *E*. *coli* and other organisms [39, 54], although larger libraries were also reported [31, 40, 55] via similar or different techniques. The activity range of the obtained library is about 100-fold, while 3- to 10-fold larger ranges have been reported in literature [37, 39]. Our smaller range could be due to the highly constrained consensus sequence used to generate the library that allows for only 64 potential sequences. We chose such consensus to maximize the probability of obtaining functional clones, since the consensus was designed according to an existing library of functional constitutive members. Our library member with highest activity was the BBa_J107100. Its activity was consistent with the popular P_LtetO1_ strong promoter and also with the activity of BBa_J23119 constitutive promoter [50], whose -35 and -10 sequences were used to design the BBa_J107100 repressible promoter. However, the random screening process did not generate members with activities higher than BBa_J107100.

The 'efficiency' of a library generation process is a controversial term, since different works provide it according to different definitions, such as the percentage of final unique members over the initial colonies, or the percentage of colonies with visible fluorescence over the total number of colonies on the transformation plate [37, 56] and apply it for diverse library generation techniques. Here, we limit the discussion to a set of features that can be compared with other library generation methods. We obtained about 10% functional unique clones out of the initially screened 90 colonies, consistent with another work [39]. However, we excluded 14 functional promoters that were twins of the 10 selected members, while in the other cited work only 5 out of 27 clones were twins [39]. This high percentage of non-unique promoters was expected, since the highly constrained consensus allowed the generation of only 64 promoters. Non-homogeneous fluorescence output was previously observed in an error prone PCR-obtained library [39], while in our work all the clones showed unimodal behaviour. Sequencing revealed that all our clones did not have heterogeneous DNA sequences, which were previously observed to be frequent in a library obtained via PCR with Taq polymerase (like in our method), probably because our protocol included a second PCR reaction with a non-degenerate primer pair to avoid the annealing of heterologous DNA strands due to nucleotide degeneration [40]. The mentioned statistics refer to the parts obtained via the first round of library construction, while the second PCR round (which generated part BBa_J107111) was not considered, since it was focused on obtaining members with a given strength range.

Regulated promoters such as the ones here constructed are widely used in biotechnology and synthetic biology research for several tasks, such as driving the expression of recombinant proteins, optimizing industrially-attractive metabolic pathways and constructing complex circuits with customized behaviour. The graded strength can be exploited to optimize the process yield, in concert with other factors such as plasmid copy number, RBS and codon optimization. The repressible behaviour of our novel components (as well as the inducible behaviour of the currently existing QS-based parts) enables the control of gene expression timing. AHL can be exogenously added or provided via other QS elements (e.g., the LuxI AHL synthase enzyme) to engineer complex, coordinated and unnatural biological functions. We expect that the herein reported library will be used in different QS-based synthetic circuits or as tunable (or constitutive) promoters in simple gene expression systems. The provided characterization data will strongly support the use of such elements in the circuit of interest according to design specifications, which can also be met with the help of mathematical models where the quantitative features of the reported promoters can be included [57]. The library of promoters described in this work has been added to the Registry and is physically available upon request.

## Supporting Information

S1 Fig Doubling times of TOP10 cells bearing the 11-member library of repressible promoters obtained via the first PCR round, tested in the constitutive (blue bars) and regulated mode without AHL (green bars). Panels A, B and C show the results in the RFP-LC, RFP-MC and GFP-MC contexts, respectively. The doubling time of TOP10 bearing the BBa_J23101 is also shown in each context. Bars represent the average value, while error bars represent the 95% confidence intervals of the mean value, computed on at least three biological replicates. Results show that no significant promoter activity-dependent trend is present and, for each context, the doubling times are comparable between constitutive and regulated mode and with the strains bearing the BBa_J23101 promoter. We only considered promoters in the regulated mode without AHL, since we found that, given a context, RFP expression never exerted a statistically significant AHL-dependent doubling time increase (evaluated by linear regression and 95% confidence intervals computation on the estimated slope).
(TIFF)

S2 Fig Hill repression curves for the initial 11 promoters from the library, with three different measurement systems. Each panel shows the curves corresponding to the promoter indicated by the number. Solid lines are the fitted curves, while circles represent the average of three experimental replicates and error bars represent the 95% confidence intervals. Blue indicates the LC-RFP context, red the MC-RFP context and green the MC-GFP context.
(TIFF)

S3 Fig Characterization results of promoter 122 in the constitutive (panel A) and regulated (panel B) mode in the RFP-LC context. A) Constitutive activity of the 12-member promoter library in the RFP-LC context, showing that promoter 122 (green bar) has successfully filled an activity gap between promoters 2 and 35. B) AHL-dependent Hill repression curve of promoter 122 in the regulated mode. Estimated parameters are reported with their 95% confidence intervals. Bars (panel A) and circles (panel B) represent the average value, while error bars represent the 95% confidence intervals of the mean value, computed on at least three biological replicates. The average doubling time of TOP10 bearing promoter 122 in the RFP-LC context was 89.7 min (95% confidence interval: 70.6–108.9 min) and 84.7 min (95% confidence interval: 63.2–106.3 min) in the constitutive and regulated (without AHL) modes, respectively. As observed in the other promoters, no significant AHL-dependent variation in doubling time was present in the regulated mode (evaluated by linear regression and 95% confidence intervals computation on the estimated slope).
(TIFF)

S4 Fig Logo sequence of the LuxR-repressible promoter library (picture generated by http://weblogo.berkeley.edu). (TIFF)

S5 Fig Study of the LuxR-AHL-dependent repressible behaviour of a set of BBa_J231xx promoters from the Anderson promoter collection. A) Constitutive and repressed activity of all available sequence-verified promoters from the Anderson promoter collection, and three additional popular promoters (P_LlacO1_, P_lacIQ_ and P_R_). Promoters were assembled in LC plasmids and were co-transformed with pLuxR-MC (including a LuxR expression cassette in the pSB3K3 vector). Blue bars represent the activity when no AHL was added to the medium, while green bars represent the repressed activity (AHL 20 μM was added to the medium). Only BBa_J23101, BBa_J23102 and BBa_J23104 showed repression and, for them, percent repression is reported. B) Repression curves for the three promoters studied. Differently from the library members, for the used AHL concentrations these promoters do not show a full-repression upon induction.
(TIFF)
