# Supplementary figures and images for: Multi-Faceted Characterization of a Novel LuxR-Repressible Promoter Library for Escherichia coli
Source: PLoS One. 2015 May 26;10(5):e0126264. doi: 10.1371/journal.pone.0126264 (PMC4444344; doi:10.1371/journal.pone.0126264)

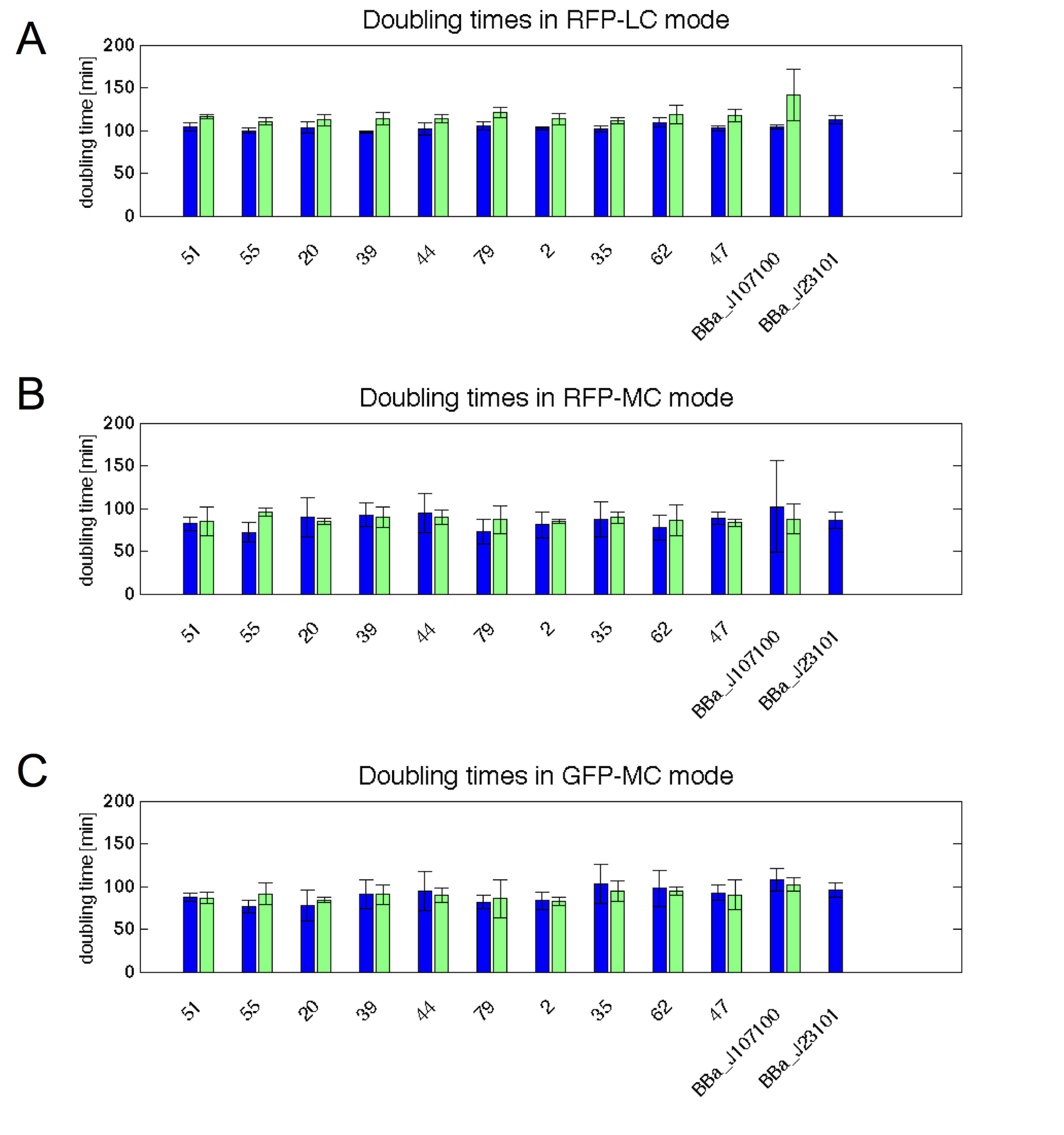

Supplement: S1 Fig — Panels A, B and C show the results in the RFP-LC, RFP-MC and GFP-MC contexts, respectively. The doubling time of TOP10 bearing the BBa_J23101 is also shown in each context. Bars represent the average value, while error bars represent the 95% confidence intervals of the mean value, computed on at least three biological replicates. Results show that no significant promoter activity-dependent trend is present and, for each context, the doubling times are comparable between constitutive and regulated mode and with the strains bearing the BBa_J23101 promoter. We only considered promoters in the regulated mode without AHL, since we found that, given a context, RFP expression never exerted a statistically significant AHL-dependent doubling time increase (evaluated by linear regression and 95% confidence intervals computation on the estimated slope). (TIFF) [file pone.0126264.s001.tiff]

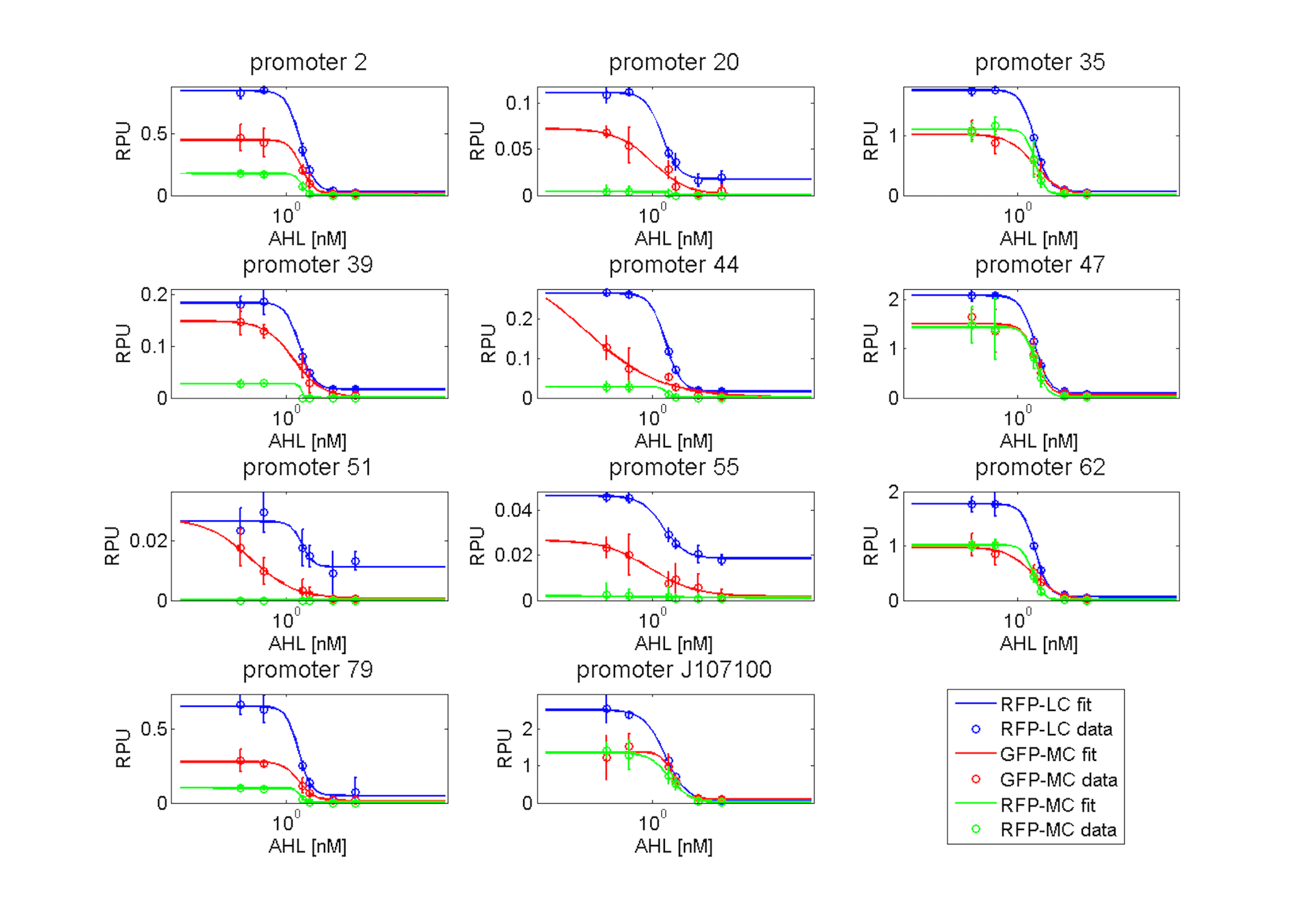

Supplement: S2 Fig — Each panel shows the curves corresponding to the promoter indicated by the number. Solid lines are the fitted curves, while circles represent the average of three experimental replicates and error bars represent the 95% confidence intervals. Blue indicates the LC-RFP context, red the MC-RFP context and green the MC-GFP context. (TIFF) [file pone.0126264.s002.tiff]

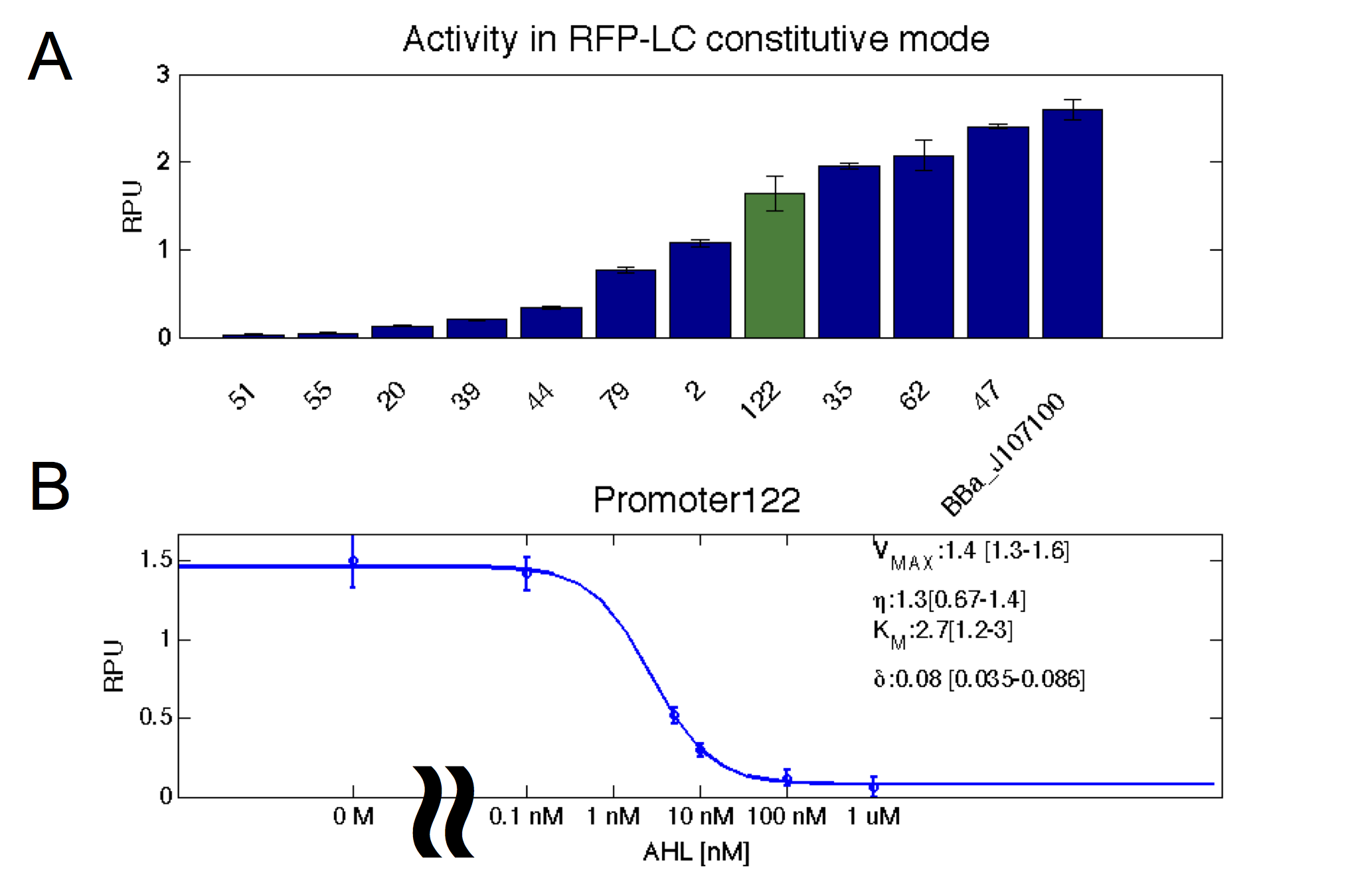

Supplement: S3 Fig — A) Constitutive activity of the 12-member promoter library in the RFP-LC context, showing that promoter 122 (green bar) has successfully filled an activity gap between promoters 2 and 35. B) AHL-dependent Hill repression curve of promoter 122 in the regulated mode. Estimated parameters are reported with their 95% confidence intervals. Bars (panel A) and circles (panel B) represent the average value, while error bars represent the 95% confidence intervals of the mean value, computed on at least three biological replicates. The average doubling time of TOP10 bearing promoter 122 in the RFP-LC context was 89.7 min (95% confidence interval: 70.6–108.9 min) and 84.7 min (95% confidence interval: 63.2–106.3 min) in the constitutive and regulated (without AHL) modes, respectively. As observed in the other promoters, no significant AHL-dependent variation in doubling time was present in the regulated mode (evaluated by linear regression and 95% confidence intervals computation on the estimated slope). (TIFF) [file pone.0126264.s003.tiff]

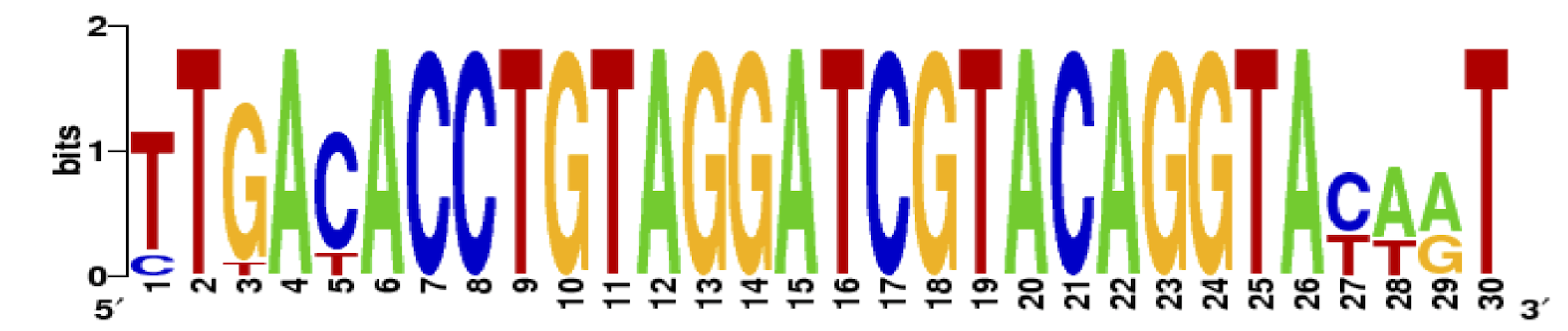

Supplement: S4 Fig — (TIFF) [file pone.0126264.s004.tiff]

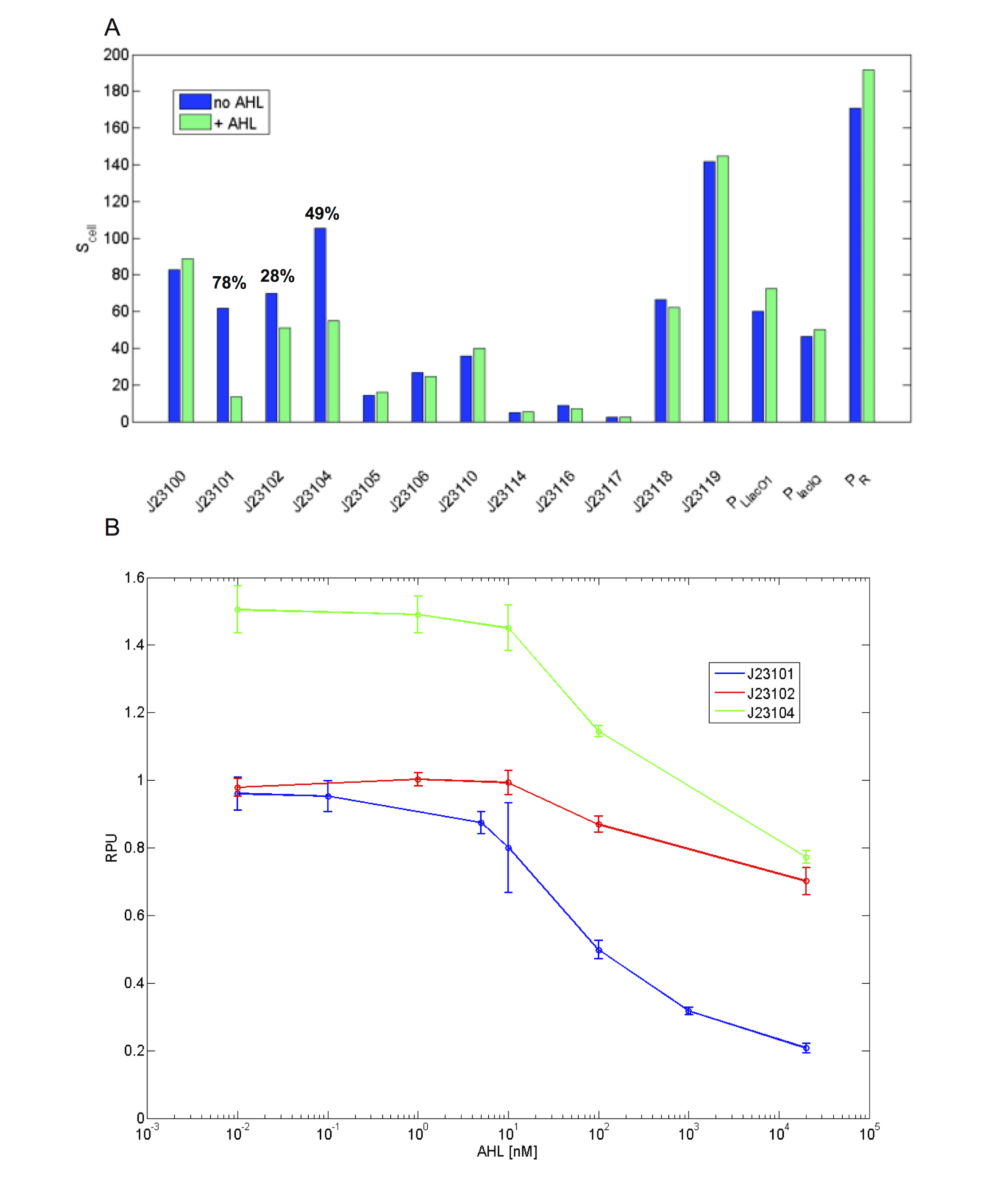

Supplement: S5 Fig — A) Constitutive and repressed activity of all available sequence-verified promoters from the Anderson promoter collection, and three additional popular promoters (PLlacO1, PlacIQ and PR). Promoters were assembled in LC plasmids and were co-transformed with pLuxR-MC (including a LuxR expression cassette in the pSB3K3 vector). Blue bars represent the activity when no AHL was added to the medium, while green bars represent the repressed activity (AHL 20 μM was added to the medium). Only BBa_J23101, BBa_J23102 and BBa_J23104 showed repression and, for them, percent repression is reported. B) Repression curves for the three promoters studied. Differently from the library members, for the used AHL concentrations these promoters do not show a full-repression upon induction. (TIFF) [file pone.0126264.s005.tiff]
